# Genetic diversity, population structure and marker-trait associations for agronomic and grain traits in wild diploid wheat *Triticum urartu*

**DOI:** 10.1186/s12870-017-1058-7

**Published:** 2017-07-01

**Authors:** Xin Wang, Guangbin Luo, Wenlong Yang, Yiwen Li, Jiazhu Sun, Kehui Zhan, Dongcheng Liu, Aimin Zhang

**Affiliations:** 1 State Key Laboratory of Plant Cell and Chromosome Engineering, National Center for Plant Gene Research, Institute of Genetics and Developmental Biology, Chinese Academy of Sciences, 1 West Beichen Road, Chaoyang District, Beijing, 100101 China; 2 University of Chinese Academy of Sciences, Beijing, 100049 China; 3 College of Agronomy/The Collaborative Innovation Center of Grain Crops in Henan, Henan Agricultural University, No. 95 Wenhua Road, Zhengzhou, 450002 China

**Keywords:** *Triticum urartu*, Genetic diversity, SSR markers, HMW-GS, Marker-trait association

## Abstract

**Background:**

Wild diploid wheat, *Triticum urartu* (*T. urartu*) is the progenitor of bread wheat, and understanding its genetic diversity and genome function will provide considerable reference for dissecting genomic information of common wheat.

**Results:**

In this study, we investigated the morphological and genetic diversity and population structure of 238 *T. urartu* accessions collected from different geographic regions. This collection had 19.37 alleles per SSR locus and its polymorphic information content (PIC) value was 0.76, and the PIC and *Nei’s* gene diversity (GD) of high-molecular-weight glutenin subunits (HMW-GSs) were 0.86 and 0.88, respectively. UPGMA clustering analysis indicated that the 238 *T. urartu* accessions could be classified into two subpopulations, of which Cluster I contained accessions from Eastern Mediterranean coast and those from Mesopotamia and Transcaucasia belonged to Cluster II. The wide range of genetic diversity along with the manageable number of accessions makes it one of the best collections for mining valuable genes based on marker-trait association. Significant associations were observed between simple sequence repeats (SSR) or HMW-GSs and six morphological traits: heading date (HD), plant height (PH), spike length (SPL), spikelet number per spike (SPLN), tiller angle (TA) and grain length (GL).

**Conclusions:**

Our data demonstrated that SSRs and HMW-GSs were useful markers for identification of beneficial genes controlling important traits in *T. urartu*, and subsequently for their conservation and future utilization, which may be useful for genetic improvement of the cultivated hexaploid wheat.

**Electronic supplementary material:**

The online version of this article (doi:10.1186/s12870-017-1058-7) contains supplementary material, which is available to authorized users.

## Background

Bread wheat (*Triticum aestivum* L.) is one of the most important crops in the world, providing about 20% of the calories consumed globally (FAOSTAT 2011; http://faostat.fao.org/). To meet the world’s growing demand for food, it is urgent to develop high yielding varieties with good end-product making quality [1]. A better understanding of the genetic basis of yield and its components is a pre-requisite, though genomic research in bread wheat remains a major challenge because of the complexity associated with its hexaploid structure and huge genome size [2, 3]. Wild diploid wheat, *T. urartu* (2n =2× =14; AA), the A-genome donor of cultivated tetraploid (2n =4× =28; genome AABB) and hexaploid wheat (2n =6× =42; AABBDD) [4], played an important role in the development and evolution of cultivated bread wheat. With the available reference genome [5], it is more feasible to exploit *T. urartu* as the reference sub-genome of common wheat, which will obviously provide considerable valuable information for the improvement of the latter.

Although *T. urartu* possesses the A genome in common with bread wheat, its genetic diversity has not been well investigated as Einkorn wheat (*T. monococcum*), another diploid progenitor [6]. Nowadays, the genetic diversity within wheat cultivars has been drastically reduced in the process of domestication and breeding [7, 8], and it is essential to apply new contributing genes for wheat improvement. As a wild diploid progenitor of hexaploid wheat, *T. urartu* harbors rich allelic diversity for numerous important traits, including agronomic characteristics, grain quality and biotic stress tolerance [9–11]. Genetic variation of *T. urartu* has been investigated using various markers such as isozyme, restriction fragment length polymorphism (RFLP), amplified fragment length polymorphism (AFLP), and random amplified polymorphic DNA (RAPD) markers [12–16]. Such genetic diversities can be exploited to elucidate the genetic basis of natural variation of important quantitative traits. Hence, more accessions widespread should be employed to provide a more comprehensive on the characteristic of the whole population.

Most agronomic traits in common wheat, such as yield and its components, dough quality and resistant characters, are controlled by multiple genes and influenced substantially by the environment, which hinders the dissection of their genetic basis [17]. Classical linkage mapping based on bi-parental populations was a conventional approach to dissect the genetic bases of complex traits [18]. Various studies have identified a set of major effect quantitative trait loci (QTL) for important agronomic traits in wheat, such as kernel weight and dough quality [17, 19–24]. As an alternative way to QTL mapping, association mapping uses diverse material to associate genetic markers with a phenotype of interest, which presents higher mapping resolution of the phenotypes at a population level [25]. It has been exploited successfully to identify genomic regions contributing to numerous traits in diverse crops, such as maize [26, 27], rice [28], sorghum [29] and soybean [30]. There is increasing interests in identifying novel marker-trait associations using association mapping in wheat [31, 32]. For example, Breseghello and Sorrells [17] found significant associations between some simple sequence repeats (SSR) markers and wheat kernel traits, including weight, length and width of kernels.

The objectives of this study were to investigate the genetic diversity, population structure and relationships among a collection of 238 *T. urartu* accessions collected from the Fertile Crescent region and to identify marker loci associated with important agronomic and grain traits. Our results would provide further insights into the utility of association mapping for marker-assisted selection and its potential application in bread wheat breeding.

## Methods

### Plant material

**Fig. 1:**
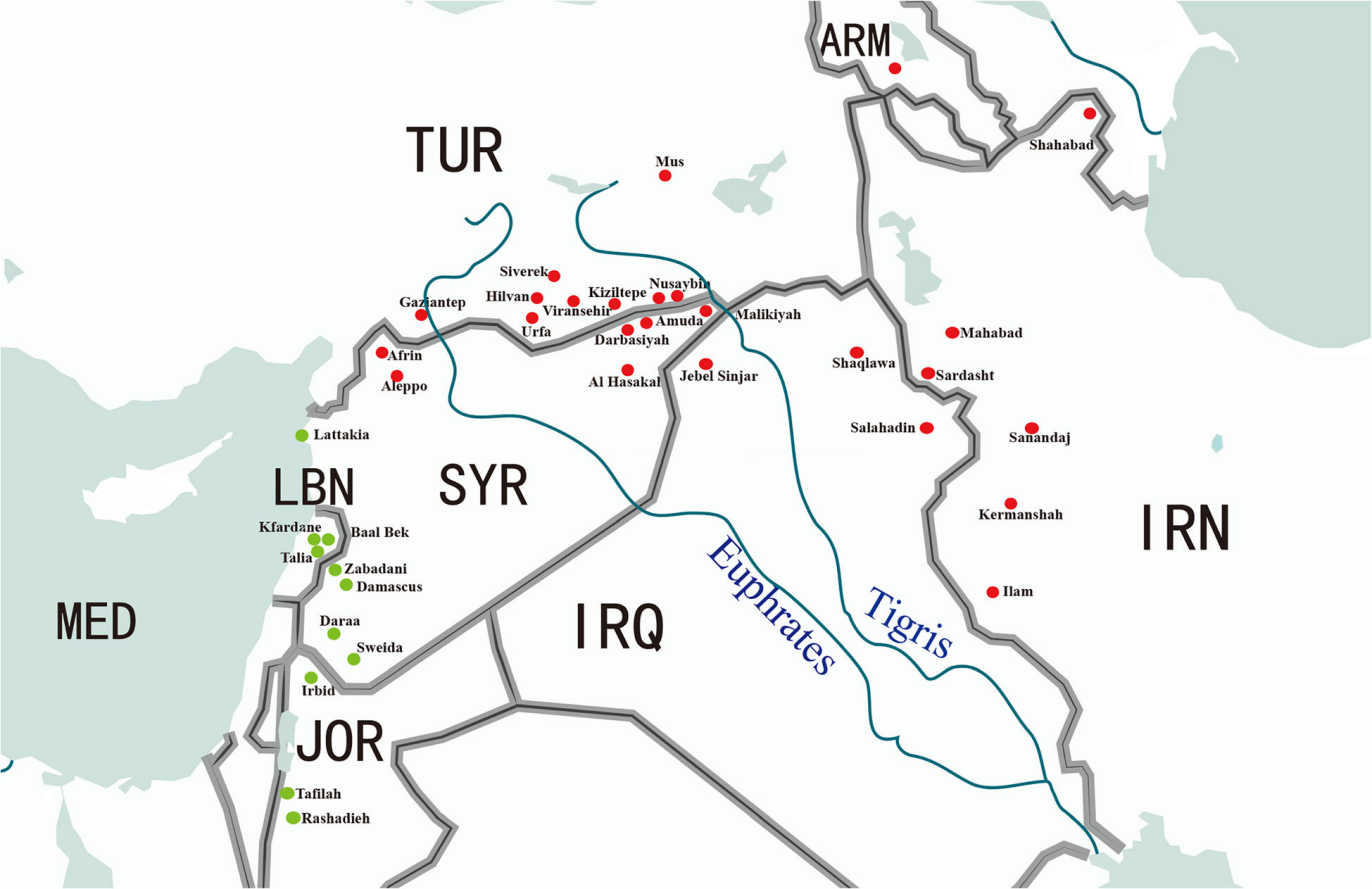
Geographic distribution of the *T. urartu* accessions used in this study. Green colors represent the locations of eastern Mediterranean coastal populations; Red colors represent the locations of Mesopotamia-Transcaucasia populations. The original map was freely downloaded from the website (http://sc.jb51.net/Web/Vector/qita/127830.html)

A total of 238 *T. urartu* accessions, which covered most of the original areas*,* were subjected to SSR and high-molecular-weight glutenin subunits (HMW-GS) analysis with SDS-PAGE. This panel was obtained from the Institute of Genetics and Developmental Biology, Chinese Academy of Sciences (IGDB, CAS), the National Small Grain collection (USDA-ARS) and the International Center for Agricultural Research in the Dry Areas (ICARDA). Among these accessions, 84 were originated from Lebanon, 80 from Turkey, 37 from Syria, 12 from Armenia, 11 from Jordan, 11 from Iran, and three from Iraq (Fig. 1; Additional file 1: Table S1).

### Field experiment and phenotyping

The collected *T. urartu* accessions were planted at the experimental station of the Institute of Genetics and Developmental Biology, Chinese Academy of Sciences, Beijing (40°5′56″N and 116°25′8″E), that of Henan Agricultural University, Zhengzhou (34°51′52″N, 113°35′45″E) and that of Dezhou Academy of Agricultural Sciences, Dezhou (37°45′69″N, 116°30′23″E) in two consecutive years (2013-2014 and 2014-2015 cropping seasons). These environments were designated as E1 (Beijing, 2013), E2 (Zhengzhou, 2013), E3 (Dezhou, 2013), E4 (Beijing, 2014), E5 (Zhengzhou, 2014), and E6 (Dezhou, 2014), respectively.

Each field trial was managed in a completely randomized block design with two replications. All the plants were grown in a single 2-m row with 40 cm between rows and 20 cm between individuals. Nine traits were evaluated and analyzed, including heading date (HD), plant height (PH), spike length (SPL), spikelet number per spike (SPLN), tiller angle (TA), grain length (GL), grain width (GW), grain length/width ratio (GLW) and thousand-grain weight (TGW). The HD was counted as days from sowing to heading, and the date of heading was subsequently recorded when half of the spikes emerged from the flag leaf in each accession. The TA was measured between the last developed tillers and the ground level with a protractor at the maximum tillering stage, while the other agronomic traits were determined on the primary tiller. After the harvest, a minimum of 200 grains from each sample was photographed on a flat-bed scanner and the images were obtained as the aerial view of the ventral side of the grains. The GL, GW and GLW were calculated using the grain analyzer software (SC-G Scanner, Wanshen Detection Technology Inc., Hangzhou, China), and the TGW was measured by the average of two 1000 kernel-weights.

### DNA isolation and nested-PCR amplification

Genomic DNA was isolated from young leaf tissue of two-week-old seedlings (one individual per accession) using the cetyl trimethyl ammonium bromide (CTAB) method [33]. DNA concentration was determined and diluted to a working solution of 50 ng/μL. Primer sequences of 62 SSR markers (Additional file 1: Table S2) used in this experiment were obtained from the GrainGenes database (https://wheat.pw.usda.gov/cgi-bin/graingenes/browse.cgi?class=marker). The nested-PCR amplifications were performed using fluorescent dye labeling system according to Schuelke [34]. In brief, amplification reactions were performed in a final volume of 15 μL, containing 3 μL template DNA, 0.2 μL forward primer with the M13 tail at its 5′-end (2.0 μM), 1.0 μL M13 primer (labeled with 6-FAM, NED, VIC or PET), 1.2 μL reverse primer (2.0 μM), 7.5 μL Mix-Taq (CWBIO, China) and 2.1 μL H_2_O (CWBIO, China). PCR was performed as follows: initial denaturing at 95 °C for 4 min, 15 cycles of 94 °C for 30 s, 60 °C for 45 s, and 72 °C for 45 s, followed by 25 cycles of 95 °C for 30s, 52 °C for 45 s, 72 °C for 45 s, and a final extension at 72 °C for 10 min. All reactions were conducted using a thermal cycler, Veriti 96 (Applied Biosystems, Foster City, USA).

Amplified PCR products were pooled and then purified with 3.0 M sodium acetate and 70% ethanol before adding HiDi-formamide. Fluorescently labeled DNA fragments were separated by capillary electrophoresis in an ABI 3730*xl*DNA Analyzer with GeneScan-500 LIZ size standard (Applied Biosystems, Foster City, CA, USA). SSR polymorphism was analyzed by GeneMapper software version 4.0 (Applied Biosystems, Foster City, CA, USA) according to the manufacturer’s instructions.

### Protein extraction and SDS-PAGE analysis

In each accession, the HMW-GS proteins were extracted from three seeds according to previous procedure [35], and SDS-PAGE was performed to fractionate the HMW-GSs using 10% (*w*/*v*) separating and 3% (*w*/*v*) stacking gels. Electrophoresis was conducted at a constant current of 18 mA at room temperature for about 8 h, and then the SDS-PAGE gels were stained overnight with Coomassie Brilliant Blue G-250 in a solution containing 20% (*v*/*v*) methanol and 10% (*v*/*v*) acetic acid. De-staining was carried out with tap water and then the gels were subjected to image capturing on a high-resolution scanner (GE ImageScanner III). The identification of HMW-GSs and alleles were based on the methodology described by [36].

### Genetic diversity and phylogenetic analysis

For 62 SSR polymorphic loci, the genetic parameters of variability were estimated via the POPGENE1.32 software [37], including number of observed (Ao) and expected (Ae) alleles per locus, observed (Ho) and expected (He) heterozygosity, *Shannon’s* information index (I), *Nei’s* Gene diversity (GD) and Polymorphism information content (PIC). The accuracy of these genotyping data was also manually checked for the scoring errors and null alleles using the Micro-Checker software [38]. All the parameters were computed for both the whole collection and the subsets considering their geographic origin and population structure category. Genetic distance and phylogenetic analyses of *T. urartu* accessions were conducted based on the Jaccard’s coefficient similarity matrix obtained from the proportion of shared SSR fragments and HMW-GS bands, then a dendrogram was drawn with NTSYSpc 2.11 program [39] using UPGMA algorithm. The fitness of the dendrogram was assessed by bootstrap analysis running 1000 replications.

### Population structure

The model-based (Bayesian) STRUCTURE version 2.3.1 was applied to identify clusters of genetically similar individuals on the basis of their genotypes [40]. The program was run five times independently for *K* value (number of subpopulations) ranging from 1 to 15, adopting the admixture model and correlated allele frequencies, with a burn-in period of 50,000 and the number of replications at 100,000. The normal logarithm of the probability was calculated against each *K* value, and the optimal number of subpopulations was determined using the *ΔK* approach described by Evanno et al. [41]. Then each *T. urartu* individuals could be assigned to the putative subpopulations according to their average membership coefficient, which was calibrated using CLUMPP [42, 43]. A nested analysis of population structure with the same software and parameters was carried out to distinguish the next level of subpopulations.

### Analysis of molecular variance (AMOVA)

Analysis of molecular variance (AMOVA) was performed to estimate the genetic variance within and among inferred populations in *T. urartu* accessions. Population differentiation was assessed by calculating pairwise *F*st values and *Nei’s* genetic distances for different regional population pairs. The threshold for statistical significance was determined by running 10,000 permutations. Principal coordinate analysis (PCoA) was also carried out based on binary genetic distance. The generated eigenvalues and accumulated percentage of the variation were applied to plot the scatter diagram of these representative accessions, with the first two principal coordinates which accounted for the highest variation. All the calculations mentioned above were implemented in the GenAlex 6.5 software [44].

### Association analysis

The markers with minor allele frequency less than 5% were removed in order to reduce false positive associations. The pairwise kinship (K matrix) among samples was generated from the program SPAGeDi [45]. Linkage disequilibrium estimates for each pair of loci and marker-trait association analysis were conducted using TASSEL 2.1 software [46], via a general linear model (GLM) and a mixed linear model (MLM). In GLM model, population structure of the *T. urartu* mapping panel was included as fixed effects, while association was estimated by simultaneous accounting of multiple levels of population structure (Q matrix) and relative kinship among the individuals (K matrix) in MLM. The significant threshold for associations between loci and traits was set at *P* < 0.01. The Bonferroni correction of multiple testing was performed based on the q value using false discovery rate (FDR, α_*c*_ = 0.05). For all associated markers, the average phenotypic effects of different alleles were estimated using the method proposed by Breseghello and Sorrells [17].

## Results

### Phenotypic variation and correlations among traits

The phenotypic data of investigated traits across the six environments, including mean values, minimum and maximum values, standard deviations and the heritability estimates (*h* ^*2*^), were calculated (Table 1). The data revealed a broad variation for all traits in these *T. urartu* accessions, e.g. PH had an average of 112.43 cm (minimum 86.10 cm and maximum 149.10 cm) with 12.23 cm standard deviation in E1, and TGW ranged from 4.29 to 18.87 g with 2.96 g standard deviation in E5. The phenotypic data for HD, PH, SPL, SPLN, GL, GW, GLW and TGW followed the normal distribution, suggesting that these traits were controlled by multiple loci. The broad-sense heritability (*h* ^*2*^) of all the traits was relatively high, ranging from 68.91% for TA (E2) to 94.05% for SPLN (E4), which confirmed that most of the phenotypic variance was genetically determined. Moreover, significant correlations coefficients among different environments were also detected, implying the less genotype x environment interactions of these traits (Additional file 1: Table S3). Depending on the collection site, all of the *T. urartu* accessions could be roughly split as Eastern Mediterranean coastal and Mesopotamia-Transcaucasia group, and the details of phenotypic performances illustrated that most of the traits investigated in this study differed greatly between the two major subsets (Additional file 1: Table S4).

**Table 1:** Phenotypic performances and distribution parameters for the investigated traits of 238 *T. urartu* accessions in six environments

Trait	Env. ^a^	Mean ^b^	Min. ^c^	Max. ^d^	SD ^e^	*h* ^*2*^ (%)^f^
HD	E1	207.43	196.00	229.00	8.57	78.18
Heading date (days)	E2	205.45	192.00	225.00	8.85	82.36
	E3	200.42	182.00	218.00	9.22	80.54
	E4	216.57	204.00	239.00	8.89	83.77
	E5	208.16	195.00	228.00	10.15	78.95
	E6	209.05	199.00	227.00	8.06	81.14
PH	E1	112.43	86.10	149.10	12.23	76.43
Plant height (cm)	E2	117.02	82.70	151.40	13.68	73.82
	E3	116.34	89.00	154.60	15.25	75.47
	E4	122.58	92.33	157.00	14.07	79.15
	E5	116.70	92.66	148.00	10.87	78.56
	E6	106.64	67.67	136.00	12.24	72.84
SPL	E1	11.10	7.38	14.92	1.62	87.22
Spike length (cm)	E2	13.02	7.02	18.43	2.31	86.61
	E3	11.19	5.00	16.03	2.05	84.89
	E4	13.09	9.00	18.67	2.07	89.27
	E5	12.52	8.38	16.90	2.00	87.33
	E6	10.63	7.30	14.80	1.65	89.06
SPLN	E1	27.29	19.20	36.00	3.76	92.73
Spikelet number/spike	E2	29.96	20.00	41.70	4.32	91.46
	E3	27.95	20.20	39.30	4.09	90.94
	E4	32.44	23.00	49.50	5.11	94.05
	E5	26.96	17.00	38.70	4.32	92.55
	E6	26.34	16.80	36.00	3.70	89.49
TA	E2	60.43	25.00	72.00	15.29	68.91
Tiller angle (°)	E4	65.14	33.00	78.00	17.60	70.87
	E5	59.32	28.00	75.00	12.59	71.53
GL	E1	6.87	5.11	8.39	0.61	90.65
Grain length (mm)	E2	7.52	6.16	8.95	0.55	87.46
	E3	6.96	5.17	8.48	0.62	89.08
	E4	7.39	5.46	8.52	0.51	90.41
	E5	7.41	6.04	8.58	0.55	88.70
	E6	7.56	6.07	9.24	0.58	87.16
GW	E1	1.58	1.05	2.07	0.21	88.78
Grain width (cm)	E2	1.80	1.32	2.28	0.22	90.52
	E3	1.58	1.12	2.04	0.21	89.67
	E4	1.74	1.22	2.23	0.19	91.33
	E5	1.78	1.41	2.11	0.20	89.29
	E6	1.77	1.33	2.24	0.17	87.06
GLW	E1	4.49	3.51	5.71	0.40	88.49
Grain length/width ratio	E2	4.26	3.50	5.19	0.41	86.95
	E3	4.49	3.76	5.55	0.41	89.42
	E4	4.37	3.55	5.47	0.42	90.13
	E5	4.26	3.69	5.05	0.35	88.35
	E6	4.37	3.69	5.04	0.38	87.29
TGW	E1	7.86	2.28	15.61	2.86	81.03
Thousand-grain weight (g)	E2	11.43	5.15	21.84	3.10	83.80
	E3	8.12	1.73	15.32	2.81	78.38
	E4	10.26	2.50	15.40	2.41	85.11
	E5	10.06	4.29	18.87	2.96	82.04
	E6	9.77	3.91	18.23	2.64	81.90

^a^ Environment: E1, E2, E3, E4, E5 and E6 represent Beijing 2013, Zhengzhou 2013, Dezhou 2013, Beijing 2014, Zhengzhou 2014 and Dezhou 2014, respectively

^b^ Mean value for *T. urartu* accessions

^c^ Minimum value among *T. urartu* accessions

^d^ Maximum value among *T. urartu* accessions

^e^ Standard deviation of each set of phenotypic data

^f^ Broad sense heritability

The correlation coefficient (*r*) analysis revealed that several traits were correlated (Additional file 1: Table S5). The highest positive correlation was detected between TGW and GW (*r* ranging from 0.68 to 0.81 in different environments, significant at *P* < 0.01), followed by TGW versus GL (0.60–0.69, *P* < 0.01), indicating that grain weight was largely influenced by grain size in *T. urartu*. In addition, significant positive correlations of HD with PH, SPL and SPLN were observed in all environments, suggesting that late-heading varieties were prone to have a high statue and large spike with many spikelets. On the other hand, HD was also negatively correlated with GL and GLW, demonstrating that a *T. urartu* accession with late heading date almost followed with small grain length and low grain length/width ratio. Notably, TA had a significant positive correlation with PH and SPLN, which reflected the tendency for prostrate type to have a high plant and a large number of spikelets.

### Genetic diversity revealed by SSR markers

To evaluate the genetic diversity of the *T. urartu* population, 62 SSR primers (loci) distributed on seven chromosomes (Table 2) were selected to perform the nested-PCR amplifications and detected with fluorescent dye labeling system. In 238 *T. urartu* accessions, a total of 1201 alleles ranging from 4 (*Xbarc138*) to 42 (*Xgwm136*) were amplified, with an average of 19.37 alleles per locus (Ao). 881 rare alleles (73.36%) were detected with the frequency lower than 5%, but none was fixed with the frequency more than 90%, resulting in an average expected allele (Ae) of 7.29. This suggested that the higher genetic variations of alleles were present in *T. urartu* accessions. Consequently, the major allele frequencies varied from 0.13 (*Xcfd15*) to 0.86 (*Xbarc206*), with the overall mean of 0.32.

**Table 2:** Diversity parameters revealed by SSR markers in 238 *T. urartu* accessions

Loci	Chromosome	Position (cM) ^a^	Repeat pattern	Size range (bp)	Ao ^b^	Ae ^c^	Gn ^d^	MAF ^e^	PIC ^f^	Ho ^g^	He ^h^	GD ^i^
*Xgwm136*	1A	14	(CT)n	224–308	42	11.43	51	0.51	0.91	0.21	0.79	0.93
*Xcfd15*	1A	23	(CT)n(TGTA)n	166–226	26	14.85	28	0.13	0.93	0.07	0.76	0.91
*Xbarc148*	1A	43	(CT)n	197–209	8	2.08	8	0.65	0.45	0	0.32	0.51
*Xgwm357*	1A	51	(GA)n	122–158	17	9.73	19	0.15	0.89	0	0.62	0.80
*Xgwm164*	1A	57	(CT)n	118–148	15	5.87	16	0.28	0.81	0	0.62	0.83
*Xcfa2129*	1A	83	(GA)n	154–206	25	9.05	25	0.23	0.91	0	0.77	0.89
*Xcfa2219*	1A	126	(GT)n	214–250	18	7.77	22	0.19	0.88	0.23	0.68	0.87
*Xbarc17*	1A	136	(TAA)n	268–325	19	5.72	19	0.32	0.86	0	0.50	0.83
*Xgwm210.1*	2A	4	(GA)n	183–187	5	1.70	5	0.73	0.35	0	0.39	0.41
*Xgwm614*	2A	10	(GA)n	120–192	36	13.25	36	0.30	0.87	0	0.70	0.88
*Xgwm328*	2A	43	(GT)n	199–212	15	8.05	26	0.28	0.87	0.36	0.72	0.88
*Xgwm249.1*	2A	59	(GA)n(GGA)n	199–238	24	6.56	24	0.24	0.83	0	0.56	0.85
*Xcfa2043*	2A	71	(GA)n	197–233	21	6.86	21	0.24	0.83	0	0.73	0.85
*Xcfa2058*	2A	76	(TC)n	252–290	24	10.34	24	0.21	0.84	0	0.73	0.90
*Xcfa2121*	2A	82	(CA)n	146–184	22	14.98	42	0.17	0.90	0.49	0.79	0.93
*Xgwm265*	2A	100	(GT)n	185–195	10	3.21	10	0.42	0.94	0	0.52	0.68
*Xgwm382.1*	2A	117	(GA)n	110–170	30	14.41	30	0.16	0.63	0	0.76	0.93
*Xcfa2086*	2A	133	(CA)n	217–284	32	11.37	32	0.22	0.93	0	0.75	0.91
*Xbarc57*	3A	0	(TTA)n	205–265	22	8.25	22	0.19	0.91	0	0.73	0.88
*Xbarc12*	3A	25	(TAA)n	167–227	20	9.08	20	0.16	0.87	0	0.72	0.89
*Xgwm369*	3A	36	(CT)nTT(CT)n	170–204	20	10.05	20	0.23	0.88	0	0.67	0.90
*Xcfa2076*	3A	61	(TG)n	184–218	14	4.02	14	0.42	0.89	0	0.55	0.75
*Xgwm674*	3A	80	(CT)nCCC(GT)n	168–178	7	1.92	7	0.66	0.42	0	0.24	0.47
*Xcfa2134*	3A	101	(TC)n	207–283	38	14.71	38	0.19	0.38	0	0.78	0.94
*Xgwm480*	3A	105	(CT)n(CA)n	176–194	12	3.71	12	0.42	0.93	0	0.46	0.73
*Xgwm247*	3A	117	(GA)n	202–214	27	6.32	27	0.29	0.69	0	0.74	0.84
*Xcfa2193*	3A	171	(GT)n	198–246	24	6.31	24	0.30	0.83	0	0.56	0.84
*Xbarc206*	4A	0	(CT)n	234–252	9	1.37	12	0.86	0.29	0.04	0.24	0.25
*Xgwm192.1*	4A	29	(CT)n	151–169	9	2.89	9	0.49	0.24	0	0.34	0.65
*Xbarc138*	4A	39	(CT)n	192–196	4	2.03	4	0.56	0.48	0	0.27	0.36
*Xgwm397*	4A	69	(CT)n	195–235	30	14.63	38	0.17	0.43	0.25	0.76	0.93
*Xgwm269.2*	4A	76	(CA)n	113–197	24	10.68	24	0.19	0.93	0	0.63	0.91
*Xcfd88*	4A	108	(CCG)n	179–182	7	1.94	7	0.61	0.90	0	0.36	0.49
*Xbarc70*	4A	187	(TATCTA)n(TCTA)n	202–274	21	8.85	21	0.43	0.38	0	0.41	0.74
*Xbarc180*	5A	12	(ATT)n	184–226	17	8.93	17	0.19	0.71	0	0.74	0.89
*Xbarc117*	5A	18	(CA)n	235–248	8	2.65	11	0.50	0.88	0.04	0.44	0.62
*Xgwm293*	5A	27	(CA)n	178–205	17	3.73	17	0.45	0.68	0	0.57	0.84
*Xbarc1*	5A	30	(TAA)n	255–297	14	2.19	14	0.38	0.61	0	0.46	0.76
*Xbarc165*	5A	35	(ATT)n	207–236	19	7.94	28	0.24	0.54	0.69	0.85	0.87
*Xbarc141*	5A	39	(GA)n	264–290	13	4.84	13	0.30	0.86	0	0.60	0.79
*Xbarc330*	5A	48	(CT)n	108–160	28	11.45	28	0.17	0.77	0	0.75	0.91
*Xbarc151*	5A	54	(CT)n	202–224	12	4.65	12	0.38	0.76	0	0.61	0.78
*Xgwm639*	5A	64	(GA)n	146–169	26	8.34	26	0.19	0.87	0	0.70	0.88
*Xgwm179*	5A	96	(GT)n	194–208	8	3.55	8	0.33	0.67	0	0.23	0.72
*Xgwm410.1*	5A	109	(CA)n	288–345	37	11.58	54	0.25	0.91	0.72	0.78	0.91
*Xgwm334*	6A	28	(GA)n	127–153	26	8.85	42	0.23	0.88	0.28	0.74	0.89
*Xbarc3*	6A	66	(CCT)n	206–256	30	9.67	30	0.33	0.84	0	0.63	0.85
*Xcfd80*	6A	84	(GA)n	165–183	11	4.04	11	0.40	0.72	0	0.53	0.75
*Xgwm132*	6A	111	(GA)n(GAA)n	112–146	20	7.01	33	0.33	0.85	0.69	0.81	0.86
*Xgwm570*	6A	118	(CT)n(GT)n	130–152	11	3.86	11	0.38	0.70	0	0.49	0.74
*Xbarc104*	6A	125	(TAA)n	174–213	13	3.11	13	0.40	0.63	0	0.54	0.78
*Xgwm427*	6A	137	(CA)n	154–228	31	12.14	31	0.17	0.91	0	0.72	0.92
*Xgwm617*	6A	140	(GA)n	106–166	11	5.28	11	0.28	0.78	0	0.42	0.75
*Xgwm471*	7A	20	(CA)n	120–186	32	9.95	32	0.27	0.89	0	0.76	0.90
*Xcfd242*	7A	39	(GTT)n(AGC)n	219–237	8	3.47	12	0.39	0.66	0.30	0.58	0.75
*Xbarc127*	7A	47	(CT)n	196–264	22	9.18	37	0.19	0.88	0.21	0.73	0.89
*Xbarc154*	7A	49	(CT)n	237–285	22	5.60	26	0.29	0.80	0.07	0.61	0.82
*Xbarc174*	7A	71	(ATT)n	170–227	21	8.85	21	0.21	0.88	0	0.55	0.89
*Xgwm276*	7A	84	(CT)n	97–129	16	4.93	20	0.37	0.77	0.06	0.61	0.80
*Xcfd20*	7A	103	(GGAA)n(CTAC)n	288–459	11	4.17	14	0.35	0.73	0.13	0.57	0.76
*Xgwm63*	7A	106	(CA)n(TA)n	157–183	16	6.61	24	0.30	0.83	0.18	0.69	0.85
*Xcfa2040*	7A	124	(CA)n	293–341	24	11.45	24	0.17	0.91	0	0.73	0.91
Mean					19.37	7.29	21.89	0.32	0.76	0.08	0.61	0.80

^a^ Indicated map positions in bread wheat according to Song et al. [48]

^b^ Number of observed alleles

^c^ Expected number of alleles

^d^ Number of genotype at each locus

^e^ Frequency of major allele

^f^ Polymorphism information content

^g^ Observed heterozygosity

^h^ Expected heterozygosity

^i^ *Nei’s* Gene diversity

We observed 18 heterozygous loci for the SSR markers assayed particularly, with the observed heterozygosity (Ho) and expected heterozygosity (He) ranged between 0.04-0.72 and 0.24-0.85, respectively. As a result, a total of 1357 genotypes were deduced with an average of 21.89 per SSR marker. According to the polymorphic information content (PIC), 53 SSR loci (85.48%) were highly informative (PIC >0.5), eight (12.90%) were reasonably informative (0.5 > PIC >0.25) and only one (1.61%) was slightly informative (PIC <0.25). Calculation of the *Nei’s* gene diversity (GD) for 62 loci demonstrated that *Xcfa2134* preserved the highest GD (GD = 0.94) and *Xbarc206* did the lowest (GD = 0.25), with the mean value of 0.80. Hardy–Weinberg equilibrium testing of these markers indicated that *T. urartu* population is not mating randomly, probably owing to the self-pollination in diploid wheat (Additional file 1: Table S6).

The panel of analyzed *T. urartu* accessions possessed high polymorphic information, covering most original areas. In order to explore and compare the variability inherent in genetic diversity, variability parameters were calculated in eight sample subsets (Table 3). The number of alleles amplified in the Mesopotamia-Transcaucasia group was higher than that in the Eastern Mediterranean coastal group (16.38 versus 11.02, *P* < 0.01), resulting in a general decrease in GD from 0.76 to 0.64. Likewise, the *Shannon’s* information indices (I) were counted as 1.97 versus 1.48 (Table 3). Beyond that, the Mesopotamia-Transcaucasia group preserved more rare alleles (frequency < 5%) (498) than the Eastern Mediterranean coastal group (377). Our data demonstrated that the Mesopotamia-Transcaucasia group had much higher genetic diversity, and these regions might be the diversity center of *T. urartu.*

**Table 3:** Genetic diversity for different *T. urartu* subsets based on 62 SSR markers

Subset of accessions	Sample Size	Ao ^a^	Ae ^b^	PIC ^c^	GD ^d^	I ^e^
All samples	238	19.37	7.29	0.76	0.80	2.04
Eastern Mediterranean coast	112	11.02	3.91	0.61	0.64	1.48
Lebanon	84	6.41	2.83	0.49	0.52	1.18
Southwestern Syria	17	7.50	4.89	0.66	0.69	1.59
Jordan	11	3.27	2.25	0.40	0.45	0.83
Mesopotamia-Transcaucasia	126	16.38	6.71	0.74	0.76	1.97
Turkey	81	11.71	5.18	0.72	0.75	1.73
Northern Syria	19	8.73	5.07	0.69	0.71	1.71
Iraq	3	2.69	2.55	0.45	0.52	1.08
Iran	11	5.53	4.28	0.66	0.69	1.41
Armenia	12	3.08	1.77	0.33	0.37	0.68

^a^ Number of observed alleles

^b^ Expected number of alleles

^c^ Polymorphism information content

^d^ *Nei’s* Gene diversity

^e^ *Shannon’s* information indices

With respect to the geographic regions analyzed separately, the subpopulation of accessions collected from Turkey exhibited the highest diversity, followed by that from Northern Syria, Southwestern Syria, Lebanon, Iran, Jordan, Armenia and Iraq, due to their differences in number of alleles and genetic diversity (Table 3). GD in each population showed a similar trend ranging from 0.75 (Turkey) to 0.37 (Armenia). The maximum and the minimum *Shannon’s* information indices (I) were observed as well in Turkish (1.73) and Armenian (0.68) accessions, respectively.

### Genetic diversity revealed by HMW-GSs

**Fig. 2:**
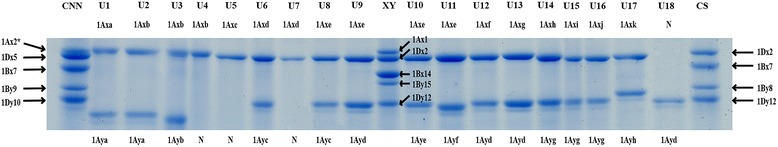
SDS-PAGE electrophoretic separations of HMW-glutenin subunits. Lanes from U1 to U18 indicate the *Glu-A1* allele band patterns in *T. urartu* accessions. Lanes CNN, XY and CS represent HMW-GS patterns in bread wheat Cheyenne, Xiaoyan 54 and Chinese Spring, respectively. N represents no allelic variants detected

The pattern of HMW-GSs from most of the *T. urartu* accessions is formed by two distinct electrophoretic moving zones, including one major 1Ax subunit zone with slower mobility and one major 1Ay subunit zone with faster mobility (Fig. 2). Among the 238 *T. urartu* accessions, the 1Ay subunit was not expressed in 69 accessions (28.99%), and only one (0.42%) was found silenced for the 1Ax subunit. All the 1Ax subunits in *T. urartu* showed faster electrophoretic mobility than the subunit 1Ax1 detected in bread wheat Xiaoyan 54 (XY), and four 1Ax subunits in 46 accessions displayed slower electrophoretic mobility than the 1Ax2^*^ present in bread wheat Cheyenne (CNN) (Fig. 2). In most *T. urartu* accessions, the electrophoretic mobility of the 1Ay subunits was faster than the 1Dy10 subunit of Cheyenne, except for one Armenian and two Turkish accessions (U17).

A total of eleven 1Ax and eight 1Ay subunits were detected, resulting in 18 HMW-GS genotypes (U1-U18) (Table 4). U1, U6 and U10 appeared exclusively in Turkish accessions, U14, U15 and U16 only in Lebanese accessions, U5 merely in southwestern Syria, and U12 and U18 were solely present in northern Syria. Concerning the frequencies of these HMW-GS genotypes, U7 was the most abundant (65 accessions, 27.31%), followed by U8 (42 accessions, 17.65%), U2 (35 accessions, 14.71%) and U14 (21 accessions, 8.82%). The remaining 14 patterns totally accounted for 31.51% of accessions, of which U1, U6 and U18 were each present in only one accession. When considering the HMW-GS locus as a co-dominant marker, its PIC and GD were 0.86 and 0.88, respectively, which were comparable with these of the SSR markers.

**Table 4:** Summary of the HMW-GS patterns analyzed by SDS-PAGE

HMW pattern	1Ax	1Ay	Original region								Accession Number	Frequency (%)
			Lebanon	Jordan	Southwestern Syria	Turkey	Northern Syria	Iraq	Iran	Armenia		
U1	a	a				1					1	0.42
U2	b	a			3	27	1	1	3		35	14.71
U3	b	b		7	1						8	3.36
U4	b	N^a^				1				1	2	0.84
U5	c	N^a^			2						2	0.84
U6	d	c	1								1	0.42
U7	d	N^a^	5	4	12	31	2		1	10	65	27.31
U8	e	c	40		1				1		42	17.65
U9	e	d				5	8	1	2		16	6.72
U10	e	e				5					5	2.10
U11	e	f				7	2	1	4		14	5.88
U12	f	d					3				3	1.26
U13	g	d				1	1				2	0.84
U14	h	g	21								21	8.82
U15	i	g	2								2	0.84
U16	j	g	15								15	6.30
U17	k	h				2				1	3	1.26
U18	N^a^	d					1				1	0.42

^a^ Not detected allelic variants

### Genetic relationships among the accessions

**Fig. 3:**
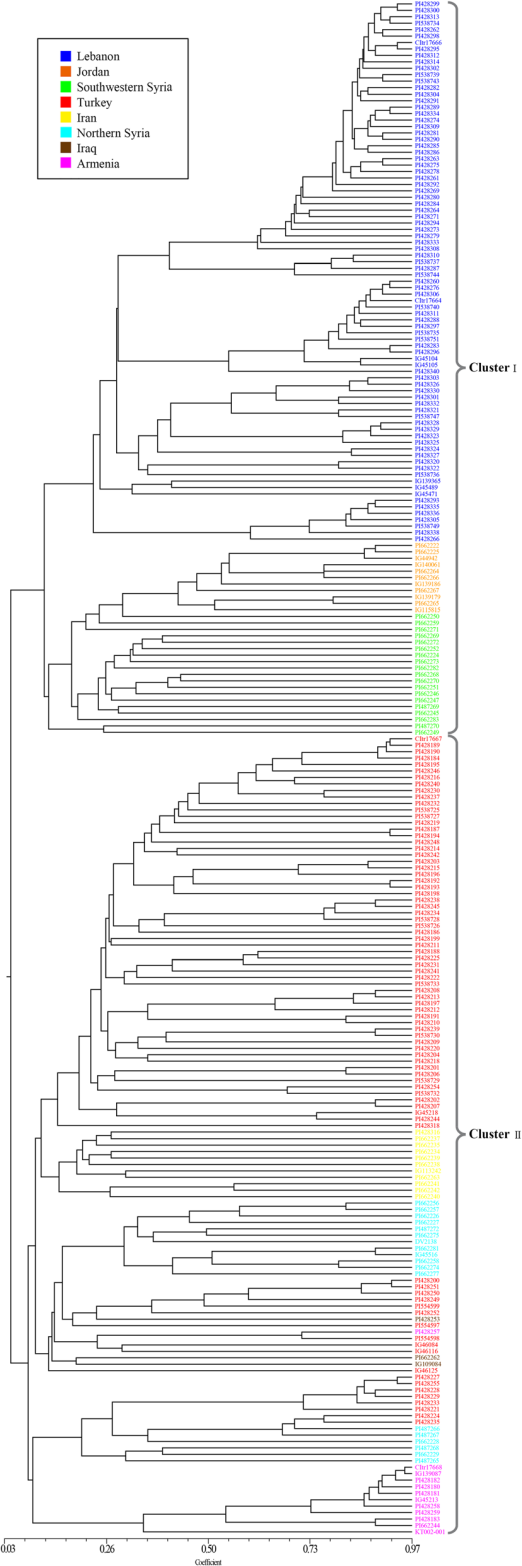
UPGMA dendrogram showing the genetic relationships among the 238 *T. urartu* accessions collected from various countries. The different subpopulations are shown in different colors

The information about genetic variation determined from SSR data combining with the SDS-PAGE analysis of HMW-GS was employed to estimate similarity matrix value. Based on Jaccard’s coefficient, an UPGMA dendrogram was constructed to reveal the genetic relationships (Fig. 3). The phylogenetic tree clearly assigned the 238 *T. urartu* accessions into two clusters: Cluster I mainly distributed in the east of Mediterranean coastal regions, including Lebanon, Jordan and southwestern Syria, and Cluster II tended to occur in the Mesopotamia and Transcaucasia regions, including Turkey, Iraq, Iran, Armenia and northern Syria. The genetic distance in the dendrogram revealed that Cluster II exhibited more diversity than Cluster I, which was consistent with its extensive geographic distribution. In Cluster I, Lebanese accessions (84) gathered tightly and were further distinguished from the other accessions, thus this cluster split into two major subclasses. Cluster II was also divided into two subclasses, one of which contained all the Iraqi and Iranian accessions (14), most of Turkish and northern Syrian accessions (84), and one Armenian accession, whereas the other one contained eight Turkish accessions, six northern Syrian accessions and eleven Armenian accessions. Typically, the sequenced accession, PI428198 (G1812) [5] grouped with most Turkish materials at the first subclass of Cluster II. The Mantel test revealed a high and significant cophenetic correlation (*r* = 0.92, *P* < 0.001), indicating a good fit between the dendrogram and its original similarity matrix. The range of similarity coefficients (0.03-0.97) showed abundant genetic variation in this collection, which is supported by the high means observed for the number of alleles per locus and the PIC values.

**Fig. 4:**
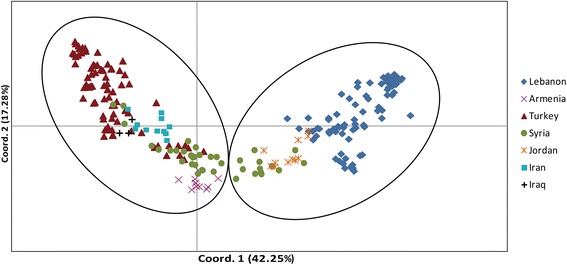
Principal coordinates analysis of 238 *T. urartu* accessions. The first and second principal coordinates account for 42.25% and 17.28% of the total variation respectively. The different colors represent the accessions of different geographical origins

Principal coordinates analysis (PCoA) was also performed in order to assess the individual differences (Fig. 4). The first two axes accounted for 42.25 and 17.28% of the genetic variation, respectively, and occupied 59.53% in total. The first coordinate clearly discriminated Eastern Mediterranean coastal accessions from Mesopotamia-Transcaucasia accessions, while the second coordinate separated the two large clusters gradually into small groups due to the latitude variation. The PCoA data confirmed the UPGMA analysis.

### Population structure

**Fig. 5:**
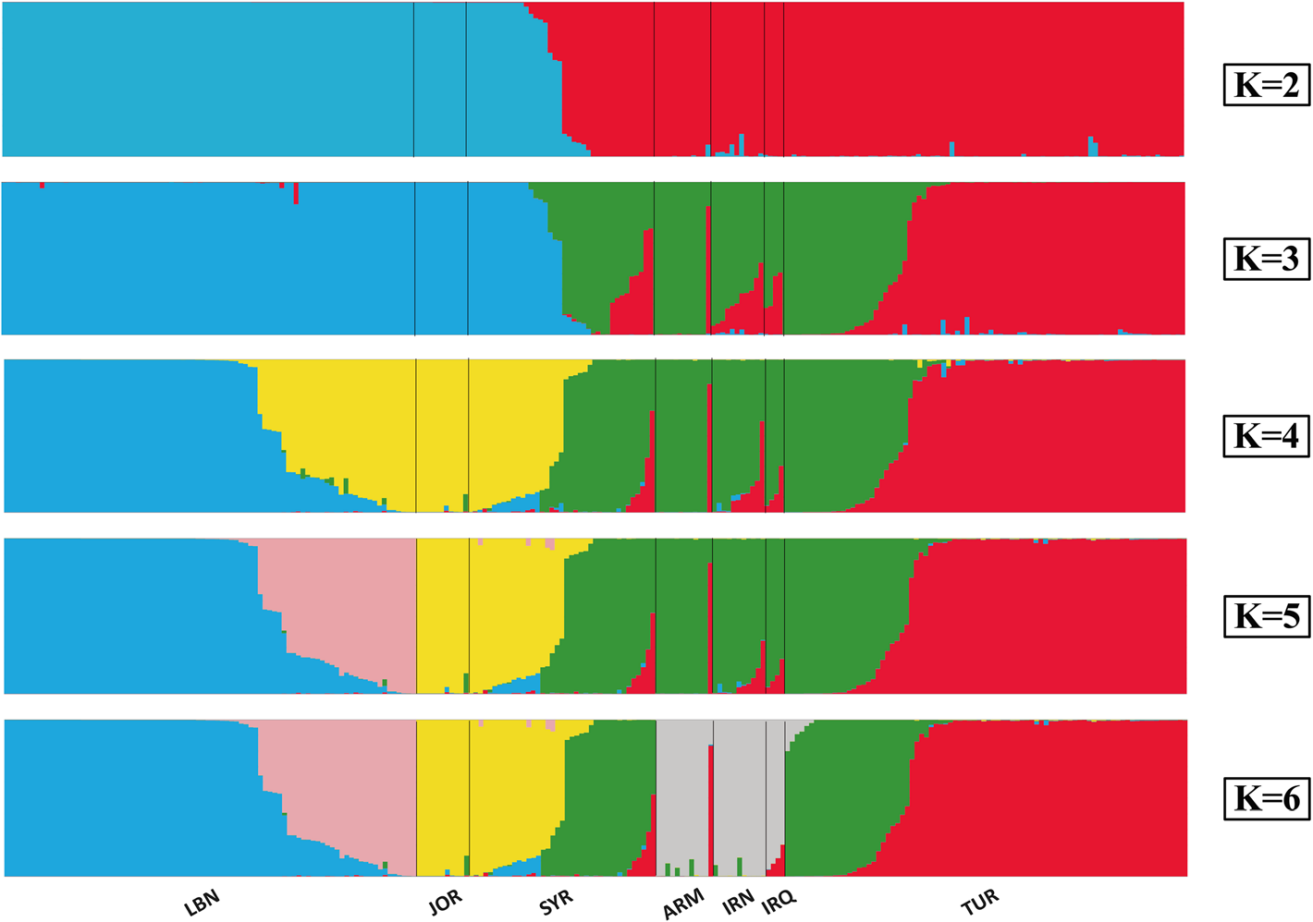
Estimated population structure from 62 nuclear SSR loci based on Bayesian clustering approaches for *K* = 2 to *K* = 6 using STRUCTURE. Each accession is represented by a vertical line. The different subpopulations are separated by a black line and shown in different colors. The bottom row indicates the geographic region. LBN, Lebanon; JOR, Jordan; SYR, Syria; ARM, Armenia; IRN, Iran; IRQ, Iraq; TUR, Turkey

In order to understand the population stratification of *T. urartu*, the model-based Bayesian cluster analysis was tested using the software program STRUCTURE (Fig. 5). The data was analyzed by successively increasing the number of subpopulations (*K*) from two to fifteen, with five independent runs for each *K* value. The CLUMPP alignment of independent solutions showed the high posterior probability in assignment of accessions among runs. At *K* = 2, we detected a division between 112 eastern Mediterranean coastal accessions (Cluster I) and 126 Mesopotamia-Transcaucasia accessions (Cluster II). At *K* = 3, Cluster II split into 61 Turkish accessions and the remainders, while 58 Lebanese accessions were segregated from the rest of Cluster I when *K* = 4. With increasing *K*, minor subpopulations from different geographic regions such as Jordan, Iran, Iraq and Armenia could be separated gradually. The optimum number of subpopulations (*K*) was identified based on lnP (D) value and the delta *K* (Δ*K*) method suggested *K* = 2 as the best fitting one in our study (Additional file 2: Fig. S1). This structure-based data was mainly consistent with the genetic relationships of the traditional clusters.

Based on AMOVA analysis, most genetic variation was detected among individuals (60.04%), followed by variation among geographic regions (18.93%), variation within individuals (12.87%) and variation among the large two Clusters (8.16%). The overall *Fst* among the Clusters and geographic regions were 0.1790 and 0.2506, respectively (*P* < 0.05). The pairwise *Fst* value, interpreted as standardized population distances between two populations, ranged from 0.0658 (between Turkish and northern Syrian populations) to 0.4419 (between Jordanian and Armenian populations). The *Nei’s* genetic distance data consisted with the *Fst* estimates, in which Turkish population showed the smallest genetic distance with northern Syrian population (0.0289), whereas the greatest genetic distance was observed between Jordanian and Armenian populations (0.7947) (Table 5).

**Table 5:** Pairwise estimates of *Fst* and *Nei’s* genetic distance between populations from different regions

Cluster	Lebanon	Jordan	Southwestern Syria	Turkey	Armenia	Iran	Iraq	Northern Syria
Lebanon		0.3060	0.3283	0.4628	0.7244	0.4340	0.6343	0.5932
Jordan	0.2044		0.0334	0.4419	0.7947	0.4399	0.6596	0.5129
Southwestern Syria	0.1590	0.1284		0.2507	0.5222	0.1944	0.4769	0.2061
Turkey	0.1813	0.2022	0.1058		0.4803	0.0312	0.1696	0.0289
Armenia	0.3290	0.4419	0.2361	0.2251		0.3852	0.4790	0.4587
Iran	0.1876	0.2131	0.1043	0.0790	0.2180		0.1731	0.0434
Iraq	0.2151	0.2343	0.1333	0.0985	0.2567	0.1081		0.0295
Northern Syria	0.1826	0.1968	0.0953	0.0658	0.2164	0.0788	0.0690	

*Nei’s* genetic distance estimates appear above the diagonal and pairwise *Fst* appears below the diagonal

### Linkage disequilibrium and marker-trait association analysis

The SSR and HWM-GS data were subjected to evaluate the linkage disequilibrium (LD) on a whole genome level. Across all the 63 loci, 1953 locus pairs were detected in the entire *T. urartu* collection, with 257 possible linked (in the same linkage groups) and 1696 unlinked locus pairs (from different linkage groups), respectively. Among these linked locus pairs, the pairwise *r* ^*2*^ values varied from 0.00 to 0.25 with a median of 0.04, and only nine (3.50%) marker pairs were at significant LD level (*r* ^*2*^ > 0.1 and *P* < 0.001), which suggested seldom LD among the analyzed loci.

The association analysis between markers and phenotypic data was carried out using GLM and MLM models in the software TASSEL. Among the 63 × 9 marker-trait comparisons, 67 significant associations (11.82%) were identified using GLM approach with Q-matrix. However, the MLM analysis reduced to 10 significant associations (1.76%) once considering K-matrix as co-variate. The Q-matrix (*K* = 2) inferred from STRUCTURE defined the ancestry coefficient of individuals in the population and the K-matrix was subjected to correct for their genetic relatedness. Besides, the averages of the phenotypic variations calculated by the two models for association mapping were 15.89% (Q) and 22.74% (Q + K), indicating that the MLM could explain more genetic variation than GLM. Henceforth, our attention will focus on associations incorporating both Q-matrix and K-matrix since they were more conservative and accurate.

Considering the MLM statistics, significant marker-trait associations for six traits were identified with six SSRs and protein marker HMW-GSs (HD: *Xcfa2193-3A*; PH: *Xgwm328-2A*; SPL: *Xgwm328-2A* and *Xgwm63-7A*; SPLN: *Xgwm328-2A*, *Xgwm63-7A* and *Xbarc138-4A*; TA: HMW-GS; GL: *Xcfa2219-1A* and *Xgwm293-5A*) in *T. urartu* accessions (Table 6). The *Xcfa2193-3A* on chromosome 3A was highly associated with HD in four environments, explaining 9.39 to 15.74% of the phenotypic variation, while *Xgwm328-2A* on chromosome 2A was closely associated with PH in all the six environments, accounting for 23.47 to 32.15% of the phenotypic variation. *Xgwm328-2A* was also simultaneously associated with SPL and SPLN in at least five environments, having the phenotypic variations of 32.43 to 37.90% and 28.42 to 38.77%, respectively. This multiple marker-trait association might be attributed to the pleiotropic effects of the genetic locus or the consequential relationship among these associated traits. *Xgwm63-7A* was significantly associated with SPL and SPLN in five environments, with the phenotypic variations of 20.67 to 27.80% and 19.24 to 23.93%, respectively, and *Xbarc138-4A* showed a stable association with SPLN in four environments, with the phenotypic variations of 14.43 to 18.18%. In particular, the HMW-GSs, encoded by *Glu-1* locus on the long arm of chromosome 1A, were associated with TA in three environments, and it explained phenotypic variations ranging from 28.18 to 34.46%. As for grain traits, *Xcfa2219-1A* and *Xgwm293-5A* were associated with grain length (GL) in five environments, and could explain 15.99 to 20.33% and 23.55 to 28.23% of the phenotypic variation, respectively.

**Table 6:** Common loci and significant markers associated with agronomic and grain shape related traits

Trait	Loci	Chromosome	*P* value^b^						*R* ^*2*^ (%)^c^					
			2014^a^			2015			2014			2015		
			E1	E2	E3	E4	E5	E6	E1	E2	E3	E4	E5	E6
HD	*Xcfa2193*	3A	4.30 × 10^−4^	0.0032	-	9.62 × 10^−4^	-	1.71 × 10^−5^	10.62	15.74	-	9.39	-	9.75
PH	*Xgwm328*	2A	1.45 × 10^−5^	1.04 × 10^−6^	0.0044	9.84 × 10^−5^	2.07 × 10^−4^	2.34 × 10^−6^	30.54	28.21	25.81	23.47	27.23	32.15
SPL	*Xgwm328*	2A	8.67 × 10^−10^	5.06 × 10^−8^	5.35 × 10^−4^	1.58 × 10^−10^	6.85 × 10^−5^	4.74 × 10^−6^	35.10	37.90	32.43	36.60	35.32	35.44
	*Xgwm63*	7A	5.19 × 10^−5^	-	6.57 × 10^−5^	3.48 × 10^−4^	0.0015	1.97 × 10^−4^	26.92	-	20.67	27.80	25.11	21.59
SPLN	*Xgwm328*	2A	0.0024	9.95 × 10^−4^	3.99 × 10^−4^	0.0072	7.07 × 10^−4^	-	34.19	38.77	33.97	28.42	35.45	-
	*Xbarc138*	4A	-	6.84 × 10^−4^	0.0027	8.74 × 10^−4^	-	0.0076	-	15.72	18.18	14.43	-	15.18
	*Xgwm63*	7A	1.24 × 10^−4^	-	9.42 × 10^−5^	2.82 × 10^−5^	0.0039	5.53 × 10^−4^	23.93	-	21.85	19.24	22.25	21.85
TA	HWM-GS	1A	-	2.39 × 10^−4^	-	0.0059	6.25 × 10^−4^	-	-	34.46	-	28.18	31.09	-
GL	*Xcfa2219*	1A	0.0050	4.45 × 10^−6^	0.0098	6.87 × 10^−5^	-	9.89 × 10^−4^	19.89	16.92	20.33	15.99	-	17.93
	*Xgwm293*	5A	2.29 × 10^−6^	0.0081	3.22 × 10^−4^	9.11 × 10^−6^	0.0035	-	28.23	26.95	27.34	24.83	23.55	-

Marker–trait association was performed with linear mixed-effects model (MLM) incorporating structure Q-matrix and kinship K-matrix in TASSEL 2.1

^a^ Missing result is represented by ‘–’ due to unavailable data

^b^ Marker–trait association is significant at *P* < 0.01 with FDR correction at αc = 0.05

^c^ *R* ^*2*^ is the percentage of phenotypic variation explained by the marker

**Fig. 6:**
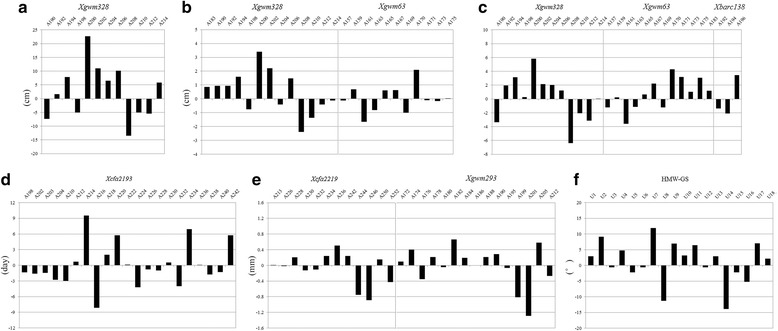
Phenotypic effect of the marker alleles at Beijing 2014 for loci significantly associated with heading date (**a**), spike length (**b**), spikelet number per spike (**c**), plant height (**d**), grain length (**e**) and tiller angle (**f**)

We further investigated the relationships between the genotype and haplotype with the phenotypic traits analyzed (Fig. 6). For HD, the 214-bp genotype of *Xcfa2193-3A*, which was observed in 10 Armenian accessions, exerted a positive effect on delaying the heading date, whereas the 202-bp genotype in 72 accessions was linked to medium heading date. Furthermore, the 200-bp allele present at *Xgwm328-2A* locus in 11 accessions was strongly associated with high values of PH, which also correlated with the increase of SPL and SPLN. In contrast, the 208-bp allele was preferentially shared by genotypes in 29 accessions with low PH, SPL and SPLN. Similarly, 55 accessions carrying the 170-bp allele at *Xgwm63-7A* produced remarkably longer SPL and more SPLN than accessions with other alleles, and the 196-bp allele at *Xbarc138-4A* could also bring more SPLN in 17 accessions than others. At *Glu-1* locus, the HMW-GS encoded U7 pattern was associated with the erect plant architecture (65 accessions), whereas the U14 pattern with the prostrate type (21 accessions). Regarding GL, the significant associations were attributed to the 224-bp allele at *Xcfa2219-1A* and the 205-bp allele at *Xgwm293-5A* being specific to genotypes with large kernel length.

## Discussion

Nowadays, the genetic variability of wheat cultivars is decreasing as a consequence of the genetic erosion of cultivated hexaploid wheat. As a wild diploid progenitor of hexaploid wheat, *T. urartu* harbors rich allelic diversity for numerous important traits, including agronomic characteristics, dough quality and biotic stress tolerance [9–11]. Thus, this wild species could be exploited in yield potential and quality improvement of bread wheat.

### Morphological and genetic diversity

In recent years, SSRs markers have been proven to be an efficient tool for molecular and genetic studies in wheat [47–49]. Many SSRs covering the A genome of common wheat were employed in diploid wheat, due to their transferability among closely related species [50]. In this work, we observed a higher level of polymorphisms in *T. urartu* using SSR markers developed in common wheat*.* A total of 1201 alleles were identified from the 62 SSR loci in the 238 accessions, with an average of 19.37 alleles per locus, which is much higher than that observed in an earlier study (19.37 vs. 8.00) of 23 *T. urartu* accessions using 25 SSRs [51]. In addition, low genetic variation of *T. urartu* has also been reported with RFLP and RAPD markers [12–14]. This is the first time that such a high level of genetic diversity was characterized in *T. urartu* using SSR markers, probably because of a relatively large number of accessions collected in a wider geographic region. Moreover, compared to other wheat species, *T. urartu* possesses higher level of genetic diversity than the A genome of tetraploid and hexaploid wheat [52, 53].

HMW-GSs are the major seed storage proteins that determine dough viscoelastic properties and bread-making quality [54, 55], which could also represent useful markers for assessment of genetic variability in wild diploid wheat collections [56, 57]. In present study, a total of eleven 1Ax and eight 1Ay subunits were detected, resulting in 18 HMW-GS combinations in 238 accessions. Even though 1Ay subunit is totally inactive in common wheat [58], 71.01% *T. urartu* accessions were found to express the 1Ay subunit, which was consistent with previous reports [56, 59, 60]. The polymorphic information content (PIC) of our collections was 0.86, and the GD value was 0.88, which suggested that genetic variation revealed by HMW-GSs was comparable with that by SSR markers, probably because of the post-translational modification of protein markers.

The genetic diversity observed in this study is well reflected by the variation in multiple biological traits. For example, PH showed a broad variation with an average of 112.43 cm ranging from 86.10 cm to 149.10 cm. All these data demonstrated that the collection of *T. urartu* possessed high genetic variation, which make them suitable for mining valuable genes based on association mapping.

### Genetic relationships and population structure

*T. urartu* species are endemic to the major geographic regions of the Fertile Crescent [61, 62]. In our study, the UPGMA dendrogram divided the diverse panel into two major subpopulations consistent with their geographic origin and ecological distribution (Fig. 1). The accessions from Eastern Mediterranean coast belonged to Clusters I, spreading from Lebanon to Jordan, of where the climate was described as poor rainfall and low temperature [63]. The levels of population differentiation in the Bekaa valley of Lebanon, southwestern Syria and the plains alongside Jordan River were low owing to high degrees of gene flow, occasional migration and cross-pollination among these accessions [15]. The other possibility is that Lebanese, southwestern Syrian and Jordan populations most probably originated from the same ancestral population, which could be inferred from the rare alleles shared by multiple loci in this study. The rest of accessions from Mesopotamia and Transcaucasia belonged to Clusters II, including Turkey, Iraq, Iran, Armenia and northern Syria. Of them, most Turkish accessions collected along the east-west road from Nusaybin to Viransehir, were in the area of South Eastern Anatolian basin characterized as mild winter and warm-dry summer [64]. The Iranian samples, located in the Zagros mountain range with a similar climatic environment, were also included in the same branch of phylogenetic tree. Other places, such as Urfa, Gaziantep and Mus of Turkey, northern Syria and part of Iraq, had a continental cold and long winter with mild summer-dry climate [64], thus, these *T. urartu* accessions were grouped together. Specially, populations originated from Armenian, Nusaybin east of Turkey and Malikiyah of northern Syria exhibited high frequencies for the rare alleles such as 127-bp at *Xgwm614-2A* and 214-bp at *Xcfa2193-3A*, which separated the populations from other regions. The restricted distribution of these alleles indicated that variation at the associated loci possibly has some adaptive significance, which was supported by previous report [14]. On the whole, genetic differentiation among the accessions was also clearly demonstrated by the first and the second principal coordinates (Fig. 4).

In order to avoid distortion of the relationships among members and the spurious association mapping between phenotype and genotype, we examined population structure of the representative *T. urartu* collection used in this study. The result separated these accessions into Eastern Mediterranean coastal and Mesopotamia-Transcaucasia populations, generally in agreement with the phylogenetic tree and PCoA analysis. However, the loss of genetic diversity and decrease of population size were detected from Cluster II to Cluster I, since bottleneck and genetic drift may generate in the duration of natural selections. Given the subdivision, accessions from Turkey and northern Syria exhibited higher diversity than others, which had also been reported previously [15, 51], and huge genetic variations were subsequently emerged among different originated populations. Our results provided inspirations for preservation and sampling of natural *T. urartu* populations in these regions.

### Marker-trait associations

Agronomic traits such as PH, HD, SPL and TA play important roles in wheat life cycle and environment adaptability, which are closely associated with the yield potential [19, 65]. Grain shape, as specified by GL, GW and GLW, is a crucial determinant of grain appearance quality and grain weight in wheat [66]. Therefore, these quantitative traits have drawn major attention in the process of wheat breading over the world. Compared to traditional QTL mapping, association mapping is less time-consuming as no segregating population needs to be developed and no segregating offspring needs to be grown [25]. Under such circumstances, a number of marker-trait associations have already been identified for significant meta-QTLs in bread wheat [67–70]. Nevertheless, a genome-wide association mapping study of agronomic traits based on elite diploid wheat germplasm is still lacking. In this study, six SSR and HMW-GS markers were detected to be highly associated with six agronomic and grain shape related traits in *T. urartu* (Table 5). The *Xcfa2193-3A* on chromosome 3A associated with HD explained >35.41% of phenotypic variation in four environments, which had also been reported in common wheat [71]. *Xgwm328-2A* and *Xgwm63-7A* associated with SPL/SPLN in our study appeared to increase both SPL and SPLN in common wheat [72, 73]. As for GL, we also detected a closely associated SSR marker *Xcfa2219-1A*, which had been reported to locate in an additive-effect QTL controlling GL in common wheat [74]. *Xgwm293* on chromosome 5A was recently characterized associated with GL and TKW in a doubled haploid (DH) mapping population [75]. Moreover, *Xbarc138-4A* for SPLN was associated with yellow rust in ITMI-mapping population [76]. The data demonstrated that these genetic regions may be conservative between *T. urartu* and hexaploid wheat, and *T. urartu* could be explored in underlying gene characterization through mapping based cloning for its relative simple genome [5]. Interestingly, the marker of HMW-GSs showed a significant association with TA after the correction of FDR. In this case, the U7 pattern containing only one 1Ax subunit was associated with the erect type, whereas the U14 pattern containing both 1Ax and 1Ay subunits was associated with the prostrate type. However, the common wheat varieties rarely express 1Ay subunit and tend to develop an erect plant, this differentiation may be due to the evolution and domestication from *T. urartu* to hexaploid wheat.

Except the phenotypic traits mentioned above, the A genome of wheat is also known to contain QTLs or genes affecting other agronomic traits, heat and drought tolerance, pathogen resistance and so on [77–79]. The allelic variation between *T. urartu* and *T. aestivum* indicates the great potential for discovery and utilization of wild diploid relatives in wheat breeding. The associations determined in this study would be very useful for marker-assisted selection (MAS) in wheat breeding programs, although more effort is needed to validate these associations in other populations. Along with the progress and wide applications of comparative genomics approaches, further work to elucidate the genetic basis of complex traits will be accelerated.

## Conclusion

Genetic diversity and population structure of 238 *T. urartu* accessions were analyzed through SSR and HMW-GS markers and their associations with phenotypes were detected. Six markers, associated with HD, PH, SPL, SPLN, TA and GL were determined, which should be beneficial to effectively exploit new genetic variations of the wild diploid *T. urartu* in yield and quality improvement of common wheat using MAS programs. Our results also provide further insights into conservation and future utilization of wild wheat resources.

## Additional files

Additional file 1: Table S1. *T. urartu* accessions used in the present. **Table S2.** List of SSR primers used for genetic diversity and association analysis. **Table S3.** Correlations coefficients for the investigated traits of 238 *T. urartu* accessions in six environments. **Table S4.** Details of phenotypic performances and ANOVA analysis of differences between the Eastern Mediterranean coastal and Mesopotamia-Transcaucasia groups. **Table S5.** Correlation coefficients between the investigated traits in 238 *T. urartu* accessions in six environments. **Table S6.** Summary of Hardy-Weinberg equilibrium testing for SSR markers used in this study. (DOCX 75 kb)

Additional file 2: Figure S1. Optimization of the number of subpopulations (*K* value) for 238 *T. urartu* accessions by the method of Delta *K* (Evanno et al. 2005). The peak represents the appropriate number of subpopulations. (TIFF 311 kb)

## Abbreviations

Ae: Number of expected alleles per locus
AFLP: Amplified fragment length polymorphism
AMOVA: Analysis of Molecular Variance
Ao: Number of observed alleles per locus
CTAB: Cetyl trimethyl ammonium bromide
FDR: False discovery rate
GD: *Nei’s* Gene diversity
GL: Grain length
GLM: General linear model
GLW: Grain length/width ratio
GW: Grain width
HD: Heading date
He: Expected heterozygosity
HMW-GS: High-molecular-weight glutenin subunit
Ho: Observed heterozygosity
I: *Shannon’s* information index
MAS: Marker-assisted selection
MLM: Mixed linear model
PCoA: Principal coordinate analysis
PH: Plant height
PIC: Polymorphic information content
QTL: Quantitative trait locus or loci
RAPD: Random amplified polymorphic DNA
RFLP: Restriction fragment length polymorphism
SDS-PAGE: Sodium dodecyl sulfate polyacrylamide gel electropheresis
SPL: Spike length
SPLN: Spikelet number per spike
SSR: Simple sequence repeat
TA: Tiller angle
TGW: Thousand-grain weight
UPGMA: Unweighted pair-group method with arithmetic means
