# Supplementary material for: Genetic diversity, population structure and marker-trait associations for agronomic and grain traits in wild diploid wheat Triticum urartu
Source: BMC Plant Biol. 2017 Jul 1;17:112. doi: 10.1186/s12870-017-1058-7 (PMC5494140; doi:10.1186/s12870-017-1058-7)
Supplement: Supplementary file 1 — T. urartu accessions used in the present. Table S2. List of SSR primers used for genetic diversity and association analysis. Table S3. Correlations coefficients for the investigated traits of 238 T. urartu accessions in six environments. Table S4. Details of phenotypic performances and ANOVA analysis of differences between the Eastern Mediterranean coastal and Mesopotamia-Transcaucasia groups. Table S5. Correlation coefficients between the investigated traits in 238 T. urartu accessions in six environments. Table S6. Summary of Hardy-Weinberg equilibrium testing for SSR markers used in this study. (DOCX 75 kb) [file 12870_2017_1058_MOESM1_ESM.docx]

## Supplementary tables

**Table S1** *T. urartu* accessions used in the present study (**Table S1.xlsx**)

**Table S2** List of SSR primers used for genetic diversity and association analysis

Table S3 Correlation coefficients for the investigated traits of 238 *T. urartu* accessions in six environments

Table S4 Details of phenotypic performances and ANOVA analysis of differences between the Eastern Mediterranean coastal and Mesopotamia-Transcaucasia groups

Table S5 Correlation coefficients between the investigated traits in 238 *T. urartu* accessions in six environments

Table S6 Summary of Hardy-Weinberg equilibrium testing for SSR markers used in this study

**Table S1** *T. urartu* accessions used in the present study (**Table S1.xlsx**)

**Table S2** List of SSR primers used for genetic diversity and association analysis

| Primer name | Primer sequences (5’→3’) | |
| --- | --- | --- |
| WMS136 | F: GACAGCACCTTGCCCTTTG | R: CATCGGCAACATGCTCATC |
| CFD15 | F: CTCCCGTATTGAGCAGGAAG | R: GGCAGGTGTGGTGATGATCT |
| BARC148 | F: GCGCAACCACAATGTATGCT | R: GGGGTGTTTTCCTATTTCTT |
| WMS357 | F: TATGGTCAAAGTTGGACCTCG | R: AGGCTGCAGCTCTTCTTCAG |
| WMS164 | F: ACATTTCTCCCCCATCGTC | R: TTGTAAACAAATCGCATGCG |
| CFA2129 | F: GTTGCACGACCTACAAAGCA | R: ATCGCTCACTCACTATCGGG |
| CFA2219 | F: TCTGCCGAGTCACTTCATTG | R: GACAAGGCCAGTCCAAAAGA |
| BARC17 | F: GCGCAACATATTCAGCTCAACA | R: TCCACATCTCGTCCCTCATAGTTTG |
| WMS210 | F: TGCATCAAGAATAGTGTGGAAG | R: TGAGAGGAAGGCTCACACCT |
| WMS614 | F: GATCACATGCATGCGTCATG | R: TTTTACCGTTCCGGCCTT |
| WMS328 | F: GCAATCCACGAGAAGAGAGG | R: CACAAACTCTTGACATGTGCG |
| WMS249 | F: CAAATGGATCGAGAAAGGGA | R: CTGCCATTTTTCTGGATCTACC |
| CFA2043 | F: CAGCCGAAGAAGGATTTCTG | R: GAGGCAGGAACTTAGGGGAG |
| CFA2058 | F: CCCATTGCCATCTCAGTCTT | R: ATAGTAGGCCCAAAGCGATG |
| CFA2121 | F: TAAATGGCCATCAAGCAATG | R: GCTTGTGAACTAATGCCTCCC |
| WMS265 | F: TGTTGCGGATGGTCACTATT | R: GAGTACACATTTGGCCTCTGC |
| WMS382 | F: GTCAGATAACGCCGTCCAAT | R: CTACGTGCACCACCATTTTG |
| CFA2086 | F: TCTACTTTCAGGGCACCTCG | R: TCTCTCCAAACCTCCCTGTAA |
| BARC57 | F: CGACCACCTCAGCCAACTTATTATGT | R: GCGGGGAGGCACATTCATAGGAGT |
| BARC12 | F: CGACAGAGTGATCACCCAAATATAA | R: CATCGGTCTAATTGTCAATGTA |
| WMS369 | F: CTGCAGGCCATGATGATG | R: ACCGTGGGTGTTGTGAGC |
| CFA2076 | F: CGAAAAACCATGATCGACAG | R: ACCTGTCCAGCTAGCCTCCA |
| WMS674 | F: TCGAGCGATTTTTCCTGC | R: TGACCGAGTTGACCAAAACA |
| CFA2134 | F: TTTACGGGGACAGTATTCGG | R: AAGACACTCGATGCGGAGAG |
| WMS480 | F: TGCTGCTACTTGTACAGAGGAC | R: CCGAATTGTCCGCCATAG |
| WMS247 | F: GCAATCTTTTTTCTGACCACG | R: ATGTGCATGTCGGACGC |
| CFA2193 | F: ACATGTGATGTGCGGTCATT | R: TCCTCAGAACCCCATTCTTG |
| BARC206 | F: GCTTTGCCAGGTGAGCACTCT | R: TGGCCGGGTATTTGAGTTGGAGTTT |
| WMS192 | F: GGTTTTCTTTCAGATTGCGC | R: CGTTGTCTAATCTTGCCTTGC |
| BARC138 | F: CTCGATTCGCCGTCAG | R: GTGGGGGAAGAAGAAACC |
| WMS397 | F: TGTCATGGATTATTTGGTCGG | R: CTGCACTCTCGGTATACCAGC |
| WMS269 | F: TGCATATAAACAGTCACACACCC | R: TTTGAGCTCCAAAGTGAGTTAGC |
| CFD88 | F: TAGGCATAGTTTTGGGCCTG | R: GGTAGAAGGAAGCTTCGGGA |
| BARC70 | F: GCGAAAAACGATGCGACTCAAAG | R: GCGCCATATAATTCAGACCCACAAA |
| BARC180 | F: GCGATGCTTGTTTGTTACTTCTC | R: GCGATGGAACTTCTTTTTGCTCTA |
| BARC117 | F: TCATGCGTGCTAAGTGCTAA | R: GAGGGCAGGAAAAAGTGACT |
| WMS293 | F: TACTGGTTCACATTGGTGCG | R: TCGCCATCACTCGTTCAAG |
| BARC1 | F: GCGATGCTTTTGCCTTGTTTCAG | R: GCGGCCCCTTTGACTCTTCATAG |
| BARC165 | F: GCGTAGAGCGGCTGTTAGTGTCAAATTA | R: GCGTTATCTCAAGTTTTGTAGCAGA |
| BARC141 | F: GGCCCATGGATAATTTTTGAAATG | R: CAATTCGGCCAAAGAAGAAGTCA |
| BARC330 | F: GCACTAAGCGCTCTTTATTTAC | R: CCTGCATCTGGTATGGAGA |
| BARC151 | F: TGAGGAAAATGTCTCTATAGCATCC | R: CGCATAAACACCTTCGCTCTTCCACT |
| WMS639 | F: CTCTCTCCATTCGGTTTTCC | R: CATGCCCCCCTTTTCTG |
| WMS179 | F: AAGTTGAGTTGATGCGGGAG | R: CCATGACCAGCATCCACTC |
| WMS410 | F: GCTTGAGACCGGCACAGT | R: CGAGACCTTGAGGGTCTAGA |
| WMS334 | F: AATTTCAAAAAGGAGAGAGA | R: AACATGTGTTTTTAGCTATC |
| BARC3 | F: TTCCCTGTGTCTTTCTAATTTTTTTT | R: GCGAACTCCCGAACATTTTTAT |
| CFD80 | F: ATAGGGGTTTTGAATCACTCC | R: TTGGATTTGCAGAGCCTTCT |
| WMS132 | F: TACCAAATCGAAACACATCAGG | R: CATATCAAGGTCTCCTTCCCC |
| WMS570 | F: TCGCCTTTTACAGTCGGC | R: ATGGGTAGCTGAGAGCCAAA |
| BARC104 | F: GCGCTTCCAAGGCTTAGAGGCT | R: CGAGCATCAATAATTGAGAAATACATAG |
| WMS427 | F: AAACTTAGAACTGTAATTTCAGA | R: AGTGTGTTCATTTGACAGTT |
| WMS617 | F: GATCTTGGCGCTGAGAGAGA | R: CTCCGATGGATTACTCGCAC |
| WMS471 | F: CGGCCCTATCATGGCTG | R: GCTTGCAAGTTCCATTTTGC |
| CFD242 | F: CCAGTTTGCAGCAGTCACAT | R: CAGACCTTAACGGGGTTGAA |
| BARC127 | F: TGCATGCACTGTCCTTTGTATT | R: AAGATGCGGGCTGTTTTCTA |
| BARC154 | F: GTAATTCCGGTTCCACTTGACATT | R: GGATGGGCAGCTTCAAGGTATGTT |
| BARC174 | F: TGGCATTTTTCTAGCACCAATACAT | R: GCGAACTGGACCAGCCTTCTATCTGTTC |
| WMS276 | F: ATTTGCCTGAAGAAAATATT | R: AATTTCACTGCATACACAAG |
| CFD20 | F: TGATGGGAAGGTAATGGGAG | R: ATCCAGTTCTCGTCCAAAGC |
| WMS63 | F: TCGACCTGATCGCCCCTA | R: CGCCCTGGGTGATGAATAGT |
| CFA2040 | F: TCAAATGATTTCAGGTAACCACTA | R: TTCCTGATCCCACCAAACAT |

Table S3 Correlation coefficients for the investigated traits of 238 *T. urartu* accessions in six environments

| Trait | Env. ^a^ | E1 ^b^ | E2 ^b^ | E3 ^b^ | E4 ^b^ | E5 ^b^ |
| --- | --- | --- | --- | --- | --- | --- |
| HD | E2 | 0.7060 |  |  |  |  |
| Heading date (days) | E3 | 0.6357 | 0.6416 |  |  |  |
|  | E4 | 0.7848 | 0.7108 | 0.6332 |  |  |
|  | E5 | 0.7539 | 0.7536 | 0.6396 | 0.7563 |  |
|  | E6 | 0.7072 | 0.6351 | 0.6285 | 0.7182 | 0.6842 |
| PH | E2 | 0.6824 |  |  |  |  |
| Plant height (cm) | E3 | 0.6846 | 0.6309 |  |  |  |
|  | E4 | 0.7682 | 0.6809 | 0.7249 |  |  |
|  | E5 | 0.6816 | 0.6113 | 0.6655 | 0.7117 |  |
|  | E6 | 0.6623 | 0.5206 | 0.6421 | 0.6791 | 0.6244 |
| SPL | E2 | 0.7163 |  |  |  |  |
| Spike length (cm) | E3 | 0.6409 | 0.6451 |  |  |  |
|  | E4 | 0.7508 | 0.6982 | 0.7598 |  |  |
|  | E5 | 0.6601 | 0.7398 | 0.6598 | 0.7591 |  |
|  | E6 | 0.6874 | 0.6710 | 0.7014 | 0.6897 | 0.7527 |
| SPLN | E2 | 0.7326 |  |  |  |  |
| Spikelet number/spike | E3 | 0.7128 | 0.7996 |  |  |  |
|  | E4 | 0.7921 | 0.8311 | 0.8458 |  |  |
|  | E5 | 0.7326 | 0.7284 | 0.7996 | 0.8311 |  |
|  | E6 | 0.7119 | 0.6278 | 0.6277 | 0.7283 | 0.6278 |
| GL | E2 | 0.5394 |  |  |  |  |
| Grain length (cm) | E3 | 0.6018 | 0.5067 |  |  |  |
|  | E4 | 0.6741 | 0.6425 | 0.6633 |  |  |
|  | E5 | 0.5291 | 0.5015 | 0.5289 | 0.6717 |  |
|  | E6 | 0.6023 | 0.5659 | 0.5216 | 0.7215 | 0.6748 |
| GW | E2 | 0.5704 |  |  |  |  |
| Grain width (cm) | E3 | 0.7111 | 0.5162 |  |  |  |
|  | E4 | 0.6484 | 0.6056 | 0.6911 |  |  |
|  | E5 | 0.5695 | 0.5640 | 0.6023 | 0.6751 |  |
|  | E6 | 0.5956 | 0.5416 | 0.6436 | 0.7200 | 0.6566 |
| GLW | E2 | 0.6151 |  |  |  |  |
| Grain length/width ratio | E3 | 0.7619 | 0.6465 |  |  |  |
|  | E4 | 0.6953 | 0.7784 | 0.6852 |  |  |
|  | E5 | 0.6205 | 0.6093 | 0.5617 | 0.6452 |  |
|  | E6 | 0.7213 | 0.6688 | 0.6696 | 0.7748 | 0.5289 |
| TGW | E2 | 0.4355 |  |  |  |  |
| Thousand-grain weight (g) | E3 | 0.5476 | 0.4843 |  |  |  |
|  | E4 | 0.6563 | 0.6653 | 0.6009 |  |  |
|  | E5 | 0.4868 | 0.5118 | 0.5270 | 0.4412 |  |
|  | E6 | 0.6394 | 0.6768 | 0.6702 | 0.5214 | 0.5590 |

^a^ Environment: E1, E2, E3, E4, E5 and E6 represent Beijing 2013, Zhengzhou 2013, Dezhou 2013, Beijing 2014, Zhengzhou 2014 and Dezhou 2014, respectively.

^b^ All the correlations between two environments of each trait are significant at *P* = 0.01.

Table S4 Details of phenotypic performances and ANOVA analysis of differences between the Eastern Mediterranean coastal and Mesopotamia-Transcaucasia groups

| Trait ^a^ | Env. ^b^ | Eastern Mediterranean coast | | | | Mesopotamia-Transcaucasia | | | | *P* - value ^g^ |
| --- | --- | --- | --- | --- | --- | --- | --- | --- | --- | --- |
|  |  | Mean ^c^ | Min. ^d^ | Max. ^e^ | SD ^f^ | Mean | Min. | Max. | SD |  |
| HD (days) | E1 | 203.81 | 196.00 | 216.00 | 4.24 | 208.69 | 199.00 | 229.00 | 5.32 | 2.28 × 10^-13**^ |
|  | E2 | 202.45 | 192.00 | 216.00 | 5.12 | 206.35 | 195.00 | 225.00 | 5.96 | 1.69 × 10^-7**^ |
|  | E3 | 197.35 | 182.00 | 211.00 | 5.98 | 201.27 | 186.00 | 218.00 | 5.09 | 1.18 × 10^-7**^ |
|  | E4 | 212.68 | 204.00 | 230.00 | 4.46 | 217.89 | 207.00 | 239.00 | 5.98 | 8.35 × 10^-13**^ |
|  | E5 | 204.75 | 195.00 | 217.00 | 5.04 | 209.40 | 198.00 | 228.00 | 5.99 | 6.16 × 10^-10**^ |
|  | E6 | 205.89 | 199.00 | 221.00 | 3.96 | 210.30 | 199.00 | 227.00 | 5.36 | 1.04 × 10^-11**^ |
| PH (cm) | E1 | 106.97 | 86.10 | 132.85 | 9.20 | 116.10 | 89.65 | 149.10 | 11.09 | 5.29 × 10^-11**^ |
|  | E2 | 108.67 | 82.70 | 135.80 | 10.06 | 123.66 | 93.40 | 151.40 | 10.95 | 6.69 × 10^-23**^ |
|  | E3 | 110.08 | 89.00 | 139.50 | 9.87 | 121.47 | 90.00 | 154.60 | 13.21 | 1.39 × 10^-12**^ |
|  | E4 | 116.49 | 92.33 | 146.00 | 10.17 | 126.70 | 97.00 | 157.00 | 13.13 | 1.86 × 10^-10**^ |
|  | E5 | 112.29 | 92.66 | 133.10 | 8.52 | 120.24 | 96.70 | 148.00 | 11.68 | 9.36 × 10^-9**^ |
|  | E6 | 102.24 | 67.67 | 128.00 | 9.93 | 108.36 | 81.40 | 136.00 | 11.22 | 1.37 × 10^-5**^ |
| SPL (cm) | E1 | 10.46 | 7.38 | 14.82 | 1.59 | 11.54 | 9.20 | 14.92 | 1.40 | 8.87 × 10^-8**^ |
|  | E2 | 11.98 | 7.02 | 17.93 | 1.91 | 13.83 | 7.00 | 18.43 | 2.18 | 3.76 × 10^-11**^ |
|  | E3 | 10.07 | 5.00 | 15.00 | 1.85 | 12.04 | 8.00 | 16.03 | 1.58 | 2.40 × 10^-16**^ |
|  | E4 | 12.11 | 9.00 | 17.67 | 1.91 | 13.81 | 10.33 | 18.67 | 1.85 | 3.20 × 10^-11**^ |
|  | E5 | 11.51 | 8.38 | 16.82 | 1.70 | 13.27 | 9.34 | 16.90 | 1.68 | 5.10 × 10^-14**^ |
|  | E6 | 10.00 | 7.30 | 14.20 | 1.29 | 11.11 | 7.90 | 14.80 | 1.62 | 2.00 × 10^-8**^ |
| SPLN | E1 | 26.63 | 19.20 | 34.00 | 2.66 | 26.71 | 20.20 | 36.00 | 3.45 | 0.0483^*^ |
|  | E2 | 28.37 | 20.00 | 39.30 | 3.03 | 30.40 | 23.00 | 41.70 | 4.35 | 5.00 × 10^-5**^ |
|  | E3 | 26.62 | 20.80 | 37.80 | 2.75 | 27.98 | 20.20 | 39.30 | 4.19 | 0.0038^**^ |
|  | E4 | 31.03 | 23.00 | 47.00 | 3.33 | 32.15 | 24.50 | 49.50 | 5.45 | 0.0390^*^ |
|  | E5 | 25.37 | 17.00 | 36.30 | 3.57 | 27.40 | 20.00 | 38.70 | 4.16 | 4.65 × 10^-5**^ |
|  | E6 | 25.29 | 16.80 | 34.00 | 2.55 | 26.42 | 20.00 | 36.00 | 4.36 | 0.0166^*^ |
| TA (°) | E2 | 43.61 | 25.00 | 72.00 | 15.55 | 52.51 | 29.50 | 81.70 | 12.36 | 1.71 × 10^-6**^ |
|  | E4 | 40.96 | 33.00 | 69.00 | 14.19 | 52.16 | 20.00 | 78.00 | 11.48 | 1.34 × 10^-10**^ |
|  | E5 | 32.34 | 28.00 | 60.00 | 12.83 | 42.85 | 13.00 | 75.00 | 11.51 | 1.93 × 10^-10**^ |
| GL (mm) | E1 | 6.68 | 5.11 | 7.99 | 0.49 | 7.08 | 5.37 | 8.39 | 0.64 | 1.91 × 10^-7**^ |
|  | E2 | 7.29 | 6.16 | 8.46 | 0.47 | 7.75 | 6.56 | 8.95 | 0.50 | 4.57 × 10^-12**^ |
|  | E3 | 6.78 | 5.27 | 7.94 | 0.52 | 7.16 | 5.17 | 8.48 | 0.67 | 3.03 × 10^-6**^ |
|  | E4 | 7.23 | 6.34 | 8.12 | 0.37 | 7.58 | 5.46 | 8.52 | 0.50 | 2.64 × 10^-9**^ |
|  | E5 | 7.28 | 6.04 | 8.26 | 0.48 | 7.60 | 6.25 | 8.58 | 0.50 | 1.11 × 10^-6**^ |
|  | E6 | 7.39 | 6.55 | 8.27 | 0.46 | 7.81 | 6.07 | 9.24 | 0.54 | 5.81 × 10^-10**^ |
| GW (cm) | E1 | 1.52 | 1.08 | 1.80 | 0.13 | 1.58 | 1.05 | 2.07 | 0.19 | 2.86 × 10^-7**^ |
|  | E2 | 1.71 | 1.32 | 2.00 | 0.12 | 1.82 | 1.48 | 2.28 | 0.15 | 1.41 × 10^-9**^ |
|  | E3 | 1.49 | 1.12 | 1.82 | 0.14 | 1.61 | 1.15 | 2.04 | 0.18 | 1.32 × 10^-8**^ |
|  | E4 | 1.67 | 1.45 | 1.89 | 0.10 | 1.74 | 1.22 | 2.23 | 0.14 | 4.79 × 10^-6**^ |
|  | E5 | 1.72 | 1.42 | 1.97 | 0.12 | 1.80 | 1.41 | 2.11 | 0.16 | 9.64 × 10^-6**^ |
|  | E6 | 1.70 | 1.33 | 1.94 | 0.11 | 1.79 | 1.44 | 2.24 | 0.13 | 3.47 × 10^-8**^ |
| GLW | E1 | 4.56 | 4.09 | 5.71 | 0.24 | 4.55 | 3.51 | 5.21 | 0.31 | 0.6772 |
|  | E2 | 4.33 | 3.66 | 5.19 | 0.26 | 4.35 | 3.50 | 5.10 | 0.30 | 0.6700 |
|  | E3 | 4.54 | 4.11 | 5.21 | 0.21 | 4.57 | 3.76 | 5.55 | 0.33 | 0.4688 |
|  | E4 | 4.42 | 3.68 | 5.14 | 0.23 | 4.46 | 3.55 | 5.47 | 0.31 | 0.2865 |
|  | E5 | 4.33 | 3.69 | 5.05 | 0.21 | 4.30 | 3.77 | 4.75 | 0.23 | 0.4087 |
|  | E6 | 4.43 | 3.84 | 5.01 | 0.19 | 4.46 | 3.69 | 5.04 | 0.27 | 0.3939 |
| TGW (g) | E1 | 6.94 | 2.28 | 12.53 | 2.11 | 8.41 | 2.90 | 15.61 | 2.79 | 8.98 × 10^-6**^ |
|  | E2 | 10.09 | 6.67 | 15.33 | 2.20 | 12.40 | 5.15 | 21.84 | 2.97 | 1.00 × 10^-10**^ |
|  | E3 | 7.25 | 2.50 | 15.32 | 2.22 | 8.55 | 1.73 | 15.11 | 2.78 | 1.02 × 10^-9**^ |
|  | E4 | 9.48 | 5.65 | 12.90 | 1.53 | 10.71 | 2.50 | 15.40 | 2.21 | 1.37 × 10^-6**^ |
|  | E5 | 9.40 | 4.29 | 14.44 | 2.36 | 10.61 | 4.34 | 18.87 | 2.88 | 4.98 × 10^-9**^ |
|  | E6 | 8.95 | 4.93 | 13.14 | 2.08 | 10.46 | 3.91 | 18.23 | 2.64 | 1.11 × 10^-6**^ |

^a^ Trait: HD, PH, SPL, SPLN, TA, GL, GW, GLW and TGW represent heading date, plant height, spike length, spikelet number/spike, tiller angle, grain length, grain width, grain length/width ratio and thousand-grain weight, respectively.

^b^ Environment: E1, E2, E3, E4, E5 and E6 represent Beijing 2013, Zhengzhou 2013, Dezhou 2013, Beijing 2014, Zhengzhou 2014 and Dezhou 2014, respectively.

^c^ Mean value for *T. urartu* accessions.

^d^ Minimum value among *T. urartu* accessions.

^e^ Maximum value among *T. urartu* accessions.

^f^ Standard deviation of each set of phenotypic data.

^g *^, *P* < 0.05; ^**^, *P* < 0.01.

Table S5 Correlation coefficients between the investigated traits in 238 *T. urartu* accessions in six environments ^a^

(E1-Beijing 2013).

| Trait^b^ | HD | PH | SPL | SPLN | PA | GL | GW | GLW |
| --- | --- | --- | --- | --- | --- | --- | --- | --- |
| PH | 0.2813^*^ |  |  |  |  |  |  |  |
| SPL | 0.4498^*^ | 0.5935^**^ |  |  |  |  |  |  |
| SPLN | 0.3376^**^ | 0.2121^*^ | 0.4140^**^ |  |  |  |  |  |
| PA | 0.2028^*^ | 0.2746^**^ | 0.1543 | 0.2187^*^ |  |  |  |  |
| GL | -0.2265^*^ | 0.2576^*^ | 0.2058^*^ | -0.2414^*^ | 0.0318 |  |  |  |
| GW | 0.1463 | 0.1243 | 0.0319 | 0.2016^*^ | 0.3146^**^ | 0.2128^*^ |  |  |
| GLW | -0.4651^**^ | -0.0299 | 0.0379 | -0.4475^**^ | -0.3186^**^ | 0.2424^*^ | -0.5392^**^ |  |
| TGW | -0.0073 | 0.1080 | 0.0672 | -0.0434 | 0.1982^*^ | 0.6207^**^ | 0.6843^**^ | -0.3996^**^ |

(E2-Zhengzhou 2013).

| Trait^b^ | HD | PH | SPL | SPLN | GL | GW | GLW |
| --- | --- | --- | --- | --- | --- | --- | --- |
| PH | 0.3281^**^ |  |  |  |  |  |  |
| SPL | 0.4437^**^ | 0.5880^**^ |  |  |  |  |  |
| SPLN | 0.7677^**^ | 0.4111^**^ | 0.5598^**^ |  |  |  |  |
| GL | -0.2723^**^ | 0.3550^**^ | 0.3238^**^ | -0.2816^**^ |  |  |  |
| GW | 0.2749^*^ | 0.1844^*^ | 0.0382 | 0.2717^**^ | 0.2517^*^ |  |  |
| GLW | -0.4770^**^ | 0.0512 | 0.1413 | -0.4765^**^ | 0.3107^**^ | -0.6329^**^ |  |
| TGW | -0.1255 | 0.1345 | 0.0963 | -0.0863 | 0.6470^**^ | 0.7342^**^ | -0.2310^*^ |

(E3-Dezhou 2013).

| Trait^b^ | HD | PH | SPL | SPLN | GL | GW | GLW |
| --- | --- | --- | --- | --- | --- | --- | --- |
| PH | 0.2435^*^ |  |  |  |  |  |  |
| SPL | 0.3202^**^ | 0.4064^**^ |  |  |  |  |  |
| SPLN | 0.4305^**^ | 0.2154^*^ | 0.5924^**^ |  |  |  |  |
| GL | -0.2647^**^ | 0.246^*^ | 0.3164^**^ | -0.2910^**^ |  |  |  |
| GW | 0.2370^*^ | 0.1721 | 0.0357 | 0.2643^*^ | 0.2686^*^ |  |  |
| GLW | -0.3286^**^ | 0.0382 | 0.1872 | -0.3840^**^ | 0.3793^**^ | -0.6470^**^ |  |
| TGW | -0.1452 | 0.1786 | 0.0678 | -0.0451 | 0.6281^**^ | 0.7943^**^ | -0.2830^*^ |

(E4-Beijing 2014).

| Trait^b^ | HD | PH | SPL | SPLN | PA | GL | GW | GLW |
| --- | --- | --- | --- | --- | --- | --- | --- | --- |
| PH | 0.3184^**^ |  |  |  |  |  |  |  |
| SPL | 0.4774^**^ | 0.5691^**^ |  |  |  |  |  |  |
| SPLN | 0.5580^**^ | 0.4065^**^ | 0.5190^**^ |  |  |  |  |  |
| PA | 0.1390 | 0.2876^*^ | 0.1782 | 0.2633^*^ |  |  |  |  |
| GL | -0.3175^**^ | 0.2692^*^ | 0.2291^*^ | -0.3425^**^ | 0.0070 |  |  |  |
| GW | 0.2567^*^ | 0.2525^*^ | 0.0092 | 0.2556^*^ | 0.2841^**^ | 0.1760 |  |  |
| GLW | -0.4281^**^ | 0.0271 | 0.1104 | -0.4621^**^ | -0.2165^*^ | 0.3354^**^ | -0.6760^**^ |  |
| TGW | -0.1092 | 0.1080 | 0.0272 | -0.0875 | 0.1967 | 0.5963^**^ | 0.7961^**^ | -0.3562^**^ |

(E5-Zhengzhou 2014).

| Trait^b^ | HD | PH | SPL | SPLN | PA | GL | GW | GLW |
| --- | --- | --- | --- | --- | --- | --- | --- | --- |
| PH | 0.2434^*^ |  |  |  |  |  |  |  |
| SPL | 0.3954^**^ | 0.4723^**^ |  |  |  |  |  |  |
| SPLN | 0.3637^**^ | 0.1801 | 0.3921^**^ |  |  |  |  |  |
| PA | 0.1332 | 0.2813^*^ | 0.1050 | 0.2753^*^ |  |  |  |  |
| GL | -0.2050^*^ | 0.2544^*^ | 0.3924^**^ | -0.2762^*^ | -0.0284 |  |  |  |
| GW | 0.1233 | 0.2020^*^ | 0.0332 | 0.2365^*^ | 0.3372^**^ | 0.3360^**^ |  |  |
| GLW | -0.2062^*^ | 0.0661 | 0.2221^*^ | -0.3064^**^ | -0.3911^**^ | 0.2320^*^ | -0.5237^**^ |  |
| TGW | -0.1441 | 0.1494 | 0.1470 | 0.0074 | 0.1433 | 0.6881^**^ | 0.8116^**^ | -0.3344^**^ |

(E6-Dezhou 2014).

| Trait^b^ | HD | PH | SPL | SPLN | GL | GW | GLW |
| --- | --- | --- | --- | --- | --- | --- | --- |
| PH | 0.2542^*^ |  |  |  |  |  |  |
| SPL | 0.2481^*^ | 0.4064^**^ |  |  |  |  |  |
| SPLN | 0.2150^*^ | 0.3795^**^ | 0.5920^**^ |  |  |  |  |
| GL | -0.2760^**^ | 0.1756 | 0.2761^**^ | -0.3520^**^ |  |  |  |
| GW | 0.1255 | 0.1868 | 0.0398 | 0.2811^*^ | 0.3893^**^ |  |  |
| GLW | -0.3958^**^ | -0.0644 | 0.1627 | -0.3662^**^ | 0.4314^**^ | -0.6084^**^ |  |
| TGW | -0.2176^*^ | 0.0973 | 0.0676 | -0.2474^*^ | 0.6361^**^ | 0.7766^**^ | -0.1301 |

^a^ ^*^, *P* < 0.05; ^**^, *P* < 0.01.

^b^ Trait: HD, PH, SPL, SPLN, TA, GL, GW, GLW and TGW represent heading date, plant height, spike length, spikelet number/spike, tiller angle, grain length, grain width, grain length/width ratio and thousand-grain weight, respectively.

**Table S6** Summary of Hardy-Weinberg equilibrium testing for SSR markers used in this study

| Loci ^a^ | DF ^b^ | ChiSq ^c^ |
| --- | --- | --- |
| *Xgwm136* | 1035 | 6548.93 |
| *Xcfd15* | 276 | 5072.61 |
| *Xbarc148* | 28 | 1666.00 |
| *Xgwm357* | 136 | 3595.39 |
| *Xgwm164* | 91 | 3004.78 |
| *Xcfa2129* | 276 | 5474.00 |
| *Xcfa2219* | 120 | 3208.10 |
| *Xbarc17* | 171 | 4284.00 |
| *Xgwm210.1* | 3 | 470.61 |
| *Xgwm614* | 630 | 8105.32 |
| *Xgwm328* | 78 | 359.10 |
| *Xgwm249.1* | 210 | 4760.00 |
| *Xcfa2043* | 171 | 4047.06 |
| *Xcfa2058* | 253 | 5236.00 |
| *Xcfa2121* | 231 | 708.92 |
| *Xgwm265* | 36 | 1904.00 |
| *Xgwm382.1* | 435 | 6632.26 |
| *Xcfa2086* | 465 | 7140.00 |
| *Xbarc57* | 210 | 4760.00 |
| *Xbarc12* | 190 | 4405.55 |
| *Xgwm369* | 153 | 4046.00 |
| *Xcfa2076* | 45 | 2107.70 |
| *Xgwm674* | 10 | 952.00 |
| *Xcfa2134* | 666 | 8330.66 |
| *Xgwm480* | 45 | 2124.22 |
| *Xgwm247* | 351 | 5976.44 |
| *Xcfa2193* | 253 | 5236.00 |
| *Xbarc206* | 15 | 952.11 |
| *Xgwm192.1* | 21 | 1428.00 |
| *Xbarc138* | 3 | 476.00 |
| *Xgwm397* | 406 | 5514.60 |
| *Xgwm269.2* | 253 | 5236.00 |
| *Xcfd88* | 21 | 818.66 |
| *Xbarc70* | 171 | 4032.82 |
| *Xbarc180* | 136 | 3808.00 |
| *Xbarc117* | 28 | 1507.33 |
| *Xgwm293* | 120 | 3434.78 |
| *Xbarc1* | 91 | 3094.00 |
| *Xbarc165* | 153 | 1172.86 |
| *Xbarc141* | 78 | 2856.00 |
| *Xbarc330* | 351 | 5927.61 |
| *Xbarc151* | 55 | 2380.00 |
| *Xgwm639* | 325 | 5950.00 |
| *Xgwm179* | 28 | 1666.00 |
| *Xgwm410.1* | 666 | 2818.87 |
| *Xgwm334* | 276 | 3083.20 |
| *Xbarc3* | 378 | 6255.80 |
| *Xcfd80* | 55 | 2380.00 |
| *Xgwm132* | 171 | 1574.76 |
| *Xgwm570* | 36 | 1904.00 |
| *Xbarc104* | 55 | 2380.00 |
| *Xgwm427* | 351 | 6188.00 |
| *Xgwm617* | 55 | 2380.00 |
| *Xgwm471* | 496 | 7378.00 |
| *Xcfd242* | 28 | 671.42 |
| *Xbarc127* | 210 | 2547.51 |
| *Xbarc154* | 171 | 3023.31 |
| *Xbarc174* | 190 | 4522.00 |
| *Xgwm276* | 78 | 2200.35 |
| *Xcfd20* | 45 | 1320.64 |
| *Xgwm63* | 91 | 2096.58 |
| *Xcfa2040* | 253 | 5236.00 |

^a^ All the markers are significant at *P* = 0.01.

^b^ Degree of freedom.

^c^ Chi-Square.
