# Supplementary figures and images for: Genetic diversity, population structure and marker-trait associations for agronomic and grain traits in wild diploid wheat Triticum urartu
Source: BMC Plant Biol. 2017 Jul 1;17:112. doi: 10.1186/s12870-017-1058-7 (PMC5494140; doi:10.1186/s12870-017-1058-7)

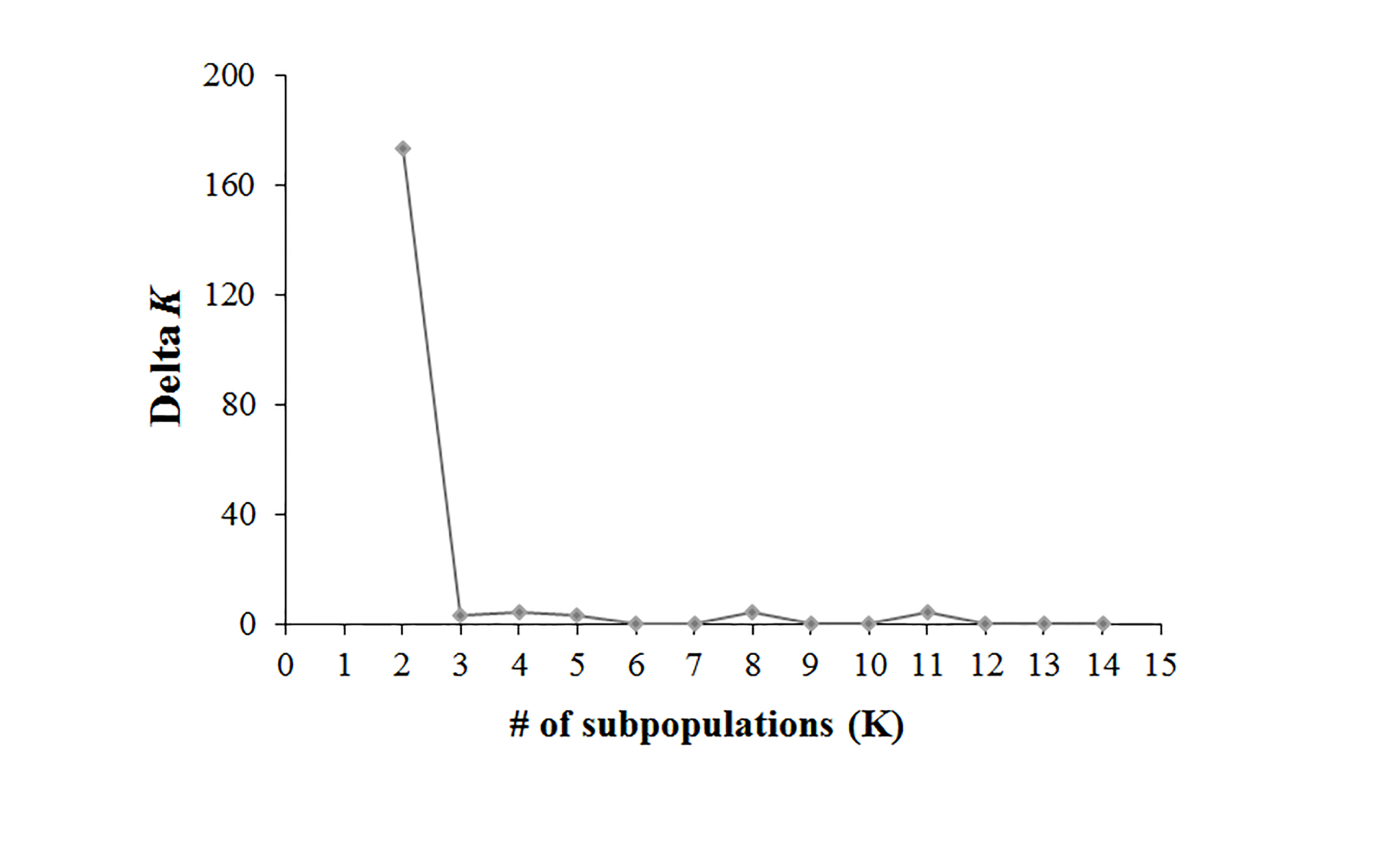

Supplement: Supplementary file 2 — Optimization of the number of subpopulations (K value) for 238 T. urartu accessions by the method of Delta K (Evanno et al. 2005). The peak represents the appropriate number of subpopulations. (TIFF 311 kb) [file 12870_2017_1058_MOESM2_ESM.tif]
